# Influence of foliar spray and post-harvest treatments on head yield, shelf-life, and physicochemical qualities of broccoli

**DOI:** 10.3389/fnut.2023.1057084

**Published:** 2023-04-17

**Authors:** Sushanta Kumar Tarafder, Mrityunjoy Biswas, Umakanta Sarker, Sezai Ercisli, Zuhal Okcu, Romina Alina Marc, Kirill S. Golokhvast

**Affiliations:** ^1^Department of Agro Product Processing Technology, Jashore University of Science and Technology, Jashore, Bangladesh; ^2^Department of Genetics and Plant Breeding, Faculty of Agriculture, Bangabandhu Sheikh Mujibur Rahman Agricultural University, Gazipur, Bangladesh; ^3^Department of Horticulture, Faculty of Agriculture, Ataturk University, Erzurum, Türkiye; ^4^Department of Gastronomy, Faculty of Tourism, Ataturk University, Erzurum, Türkiye; ^5^Department of Food Engineering, Faculty of Food Science and Technology, University of Agricultural Sciences and Veterinary Medicine, Cluj-Napoca, Romania; ^6^Siberian Federal Scientific Center of Agrobiotechnology RAS, Krasnoobsk, Russia

**Keywords:** pre-harvest foliar spay, texture, color, antioxidants, ascorbic acid, head yield, total phenol content, post-harvest packaging

## Abstract

Rapid senescence is the key factor in the deterioration of post-harvest shelf-life in broccoli heads. This study evaluates the head yield and its related traits, and physicochemical attributes of broccoli under four foliar sprays of mineral nutrients (B, Zn, Mo, and B + Zn + Mo) with control. The interaction effects of shelf-life and physicochemical attributes of broccoli for these five pre-harvest and five post-harvest storage treatments (LDP bag, HDP vacuum pack, 2% eggshell powder solution, 2% ascorbic acid, and control) both at cold storage and room temperature were evaluated with three replications. The significantly higher marketable head yield of 28.02 t ha^−1^, maximum gross return [(Bangladesh Taka (BDT 420300 ha^−1^)], net return (BDT 30565 ha^−1^), and maximum benefit–cost ratio (BCR) of 3.67 were obtained from the pre-harvest foliar application of B + Zn + Mo in broccoli. Pre-harvest foliar spray of combined nutrient B + Zn + Mo and post-harvest treatment high-density polyethylene (HDP, 15 μm) vacuum packaging efficiently improve post-harvest physicochemical attributes, viz., compactness, green color, texture, carbohydrates, fats, energy, antioxidants, vitamin C, and total phenols in broccoli head compared to the rest of the treatment combinations. In addition, this treatment combination also confirmed a maximum shelf-life of 24.55 days at cold storage [relative humidity (RH) 90–95% and 4°C] and 7.05 days at room temperature (RH 60–65% and 14–22°C) compared to the rest of the treatment combinations. Therefore, we recommend a pre-harvest foliar spray of combined nutrient elements B + Zn + Mo and an HDP (15 μm) vacuum post-harvest packaging for the maximum benefits for both farmers and consumers to get the best head yield, anticipated physicochemical attributes, and maximum shelf-life of broccoli.

## Introduction

Vegetables are low-cost sources of essential minerals, proteins (1–3), vitamins, dietary fiber (4, 5), phenols, flavonoids (6, 7), colorants with strong antiradical potential (8–10) that contribute to human nutrition, palatability, constipation (11–13), and health promotion (14). Broccoli (*Brassica oleracea* var. Italica) has been referred to as a high-quality food. It is a high-value excellent commercial crop under the *Brassicaceae* family and contains glucobrassicin, glucoraphanin, neoglucobrassicin, progoitrin, glucoiberin, 4-methoxyglucobrassicin, glucoalyssin, and gluconasturtiin (15). An aliphatic glucosinolate, glucoraphanin is the most copious in heads of broccoli; nevertheless, 3-methylsulfinyl propyl glucosinolate (glucoiberin), glucoerucin, and glucobrassicin are also present in it (16). It is also a good source of vitamins (A, C, and K), isothiocyanates, folates, dietary phenolic compounds, fibers, and essential mineral nutrients. The bioactive compounds and nutritional values are beneficial for the inhibition of carcinogenic, obesity, and cardiovascular ailments (17). It possesses antioxidants that promote many aspects of health (18, 19). Broccoli is best known for its low glycemic index (GI = 10), a functional food that reduces hepatic glucose production and improves glucose control in patients with type-2 diabetes (20).

In the year 2019, the total production of broccoli in the world was 27 million tons. The majority (73%) of the world's broccoli was produced in China and India. Mexico, the USA, Spain, Turkey, Italy, Bangladesh, France, and Poland produced the rest (27%) of the world's broccoli (21). Advanced growers in Bangladesh are very enthusiastic about growing and spreading the farming area for broccoli due to its high nutritional value and high price.

Fertilizer management is crucial for high-quality head production in broccoli to achieve maximum return (22). The growers in Bangladesh are applying chemical fertilizers scattered to obtain a better head yield of broccoli without the utilization of necessary organic manures, which leads to soil health deterioration. As a result, it reduces the quality and shelf-life of broccoli (23). In Bangladesh, head yield and quality of broccoli are drastically decreased due to insufficient use of essential mineral nutrients, such as B, Zn, and Mo (24). Zn, Mo, and B spray in foliage meaningfully increases growth, yield, development, quality, and height, reducing hollow stem infestation in broccoli (25–27). Zn directly relates to photosynthesis, respiration, and carbon metabolism and stimulates tryptophan (precursor of IAA) synthesis, and increases growth, shoot production, and height. B significantly involves in the occurrence of cell division, ion absorption, fruit development, cell wall formation, translocation of sugars, carbohydrates transportation and metabolism, water relations, and hormone activation (26, 27). Hence, the meticulous application of these essential mineral nutrients helps to increase the growth, yield, development, and quality of broccoli (26, 28). It is assumed that the application of soil test-based chemical fertilizers along with the foliar spray of mineral nutrients in broccoli during cultivation enhances head yield.

Temperature management is the most important activity to extend the shelf-life and indirectly delay losses of quality parameters of broccoli during post-harvest storage. Yellowing and wilting of broccoli heads indicate deterioration which leads to reduced market value owing to consumers' choice (29). Low-density polyethylene (LDP) and high-density polyethylene (HDP) are viable post-harvest treatment technologies to extend the shelf-life of broccoli which slow down the respiration rate and ethylene production, keep color intact, and maintain texture, flavor, and nutritive values (30). Polyethylene bag protects the color, compactness, and texture of broccoli due to the combined effect of the fluctuated atmosphere, high humidity, and low temperature (31). HDP vacuum pack in cold storage conditions (storage with 95% relative humidity (RH) at 4°C) is a fruitful technique to continue the shelf-life and quality of broccoli (32).

The literature has shown that individual foliar spray of Zn during pre-harvest displayed the highest stalk length, plant spread, root length, ascorbic acid, and B increased the TSS, head yield and carbohydrate content. However, Mo increased the reducing sugar (33). Thus, foliar application of each mineral nutrient may be a promising method for maximizing broccoli productivity and growth (25–27, 34). Similarly, polyethylene bags (HDP) enhance the efficiency in post-harvest quality and shelf-life of broccoli (29, 35), and eggshells can be used for the preservation of quality fresh vegetables and fruits (36). Post-harvest dipping of fruit in ascorbic acid solution may be used as a preferential method for improvement of shelf-life and increasing the keeping quality intact for a long time (37). It is hypothesized that Zn, Mo, and B spray in foliage and post-harvest treatments using low-cost technologies such as LDP (35 μm) bag, HDP vacuum pack (15 μm), 2% eggshell powder, and 2% ascorbic acid solution might be an effective way to maintain the quality and improve shelf-life of broccoli. Therefore, this study emphasizes using low-cost technologies such as LDP (35 μm) bag, HDP vacuum pack (15 μm), 2% ascorbic acid, and 2% eggshell powder solution in post-harvest stage and Zn, Mo, and B spray in foliage to improve physicochemical attributes and retain the shelf-life of broccoli. Although a few pre-harvest studies of some minerals on morphological traits are available, the pre-harvest effect of these elements on physicochemical traits is scarce. This is the first report regarding the in-depth study of broccoli on pre-harvest utilization of individual and combined Zn, Mo, and B in combination with post-harvest treatment materials under normal and cold storage facilities. Therefore, to fill the lacuna, the investigation was set up to take an elaborative study on the effects of combined mineral nutrients sprayed in foliage during pre-harvest and post-harvest application of different densities of 2% ascorbic acid solution, 2% eggshell powder, and polythene packaging, on the yield, shelf-life, and physicochemical attributes of broccoli.

## Materials and methods

### Location of the experimental site and duration

The study was carried out during the Rabi seasons (October to March) of 2020–2021 and 2021–2022 at a farmer's field in Palashi of Manirampur Upazila under Jashore district, Bangladesh. It is located at 23°1 0′N″ latitude and 89° 14′0″ E longitude. The altitude of the location is 9 m above the mean sea level. The experimental site is a medium-high land with loamy soil. Physical and chemical properties of the experimental plots of topsoil in 0-15 cm depth were analyzed by the soil testing laboratory, Soil Resources Development Institute (SRDI), Jashore. Details are presented in Supplementary Table S1.

### Details of treatments

The sources of mineral nutrients taken are boric acid (H_3_BO_3_) for B, Zn sulfate (ZnSO_4_) for Zn, and ammonium heptamolybdate tetrahydrate [(NH_4_)_6_Mo_7_O_2_.4H_2_O] for Mo with a single concentration of 0.40% each separately and combined along with control. The details of treatments are as follows: T_1_ = control, T_2_ = B, T_3__=_ Zn, T_4__=_ Mo and T_5__=_ B + Zn + Mo, 0.40%, respectively.

### Preparation of mineral nutrients solutions and spraying

According to treatment, fresh solutions of mineral nutrients; boric acid (H_3_BO_3_) for B, Zn sulfate (ZnSO_4_) for Zn, and ammonium heptamolybdate tetrahydrate [(NH_4_)_6_Mo_7_O_2_.4H_2_O] for Mo were prepared meticulously just before spraying. To make a 1-L solution of 0.40% concentration of each mineral nutrient, 4 g amount of each mineral nutrient was taken, respectively and weighed carefully, and dissolved in 1 L of distilled water separately. The solution was thoroughly mixed until homogeneous. According to treatment, prepared fresh solutions of mineral nutrients were applied as a foliar spray to run off stage. The first spraying was done 15 days after transplanting, and subsequently, the second, third, and fourth spraying was done at 30, 45, and 60 days after transplanting. The spraying was done in the afternoon (~4.00–4.30 P.M.) with a manual knapsack sprayer to wet the whole leaf completely until runoff. The sprayer was washed with water properly before and after spraying every solution to avoid any kind of contamination. All necessary precautions were taken into account during the spraying of chemicals.

### Design and layout of the experiment

Head yield and physicochemical attributes of broccoli were determined using a randomized complete block design (RCBD) with three replications and five treatments (T_1__=_ Control, T_2__=_ B, T_3__=_ Zn, T_4__=_ Mo, and T_5__=_ B + Zn + Mo, 0.40%, respectively). However, the shelf-life and physicochemical parameters of broccoli were also determined using a complete randomized design (CRD) in three replicates considering each of three factors; Factor 1: application of foliar mineral spray (T_1__=_ Control, T_2__=_ B, T_3__=_ Zn, T_4__=_ Mo and T_5__=_ B + Zn + Mo, 0.40%, respectively); Factor 2: post-harvest treatments at room temperature (i) LDP bag, (ii) HDP vacuum pack, (iii) 2% eggshell powder solution, (iv) 2% ascorbic acid solution, and v. control) and Factor 3: post-harvest treatments at cold storage condition (i) LDP bag, (ii) 2% eggshell powder solution, (iii) HDP vacuum pack, (iv) control, and (v) 2% ascorbic acid solution).

### Sources of planting materials

Green Crown (hybrid) variety of broccoli was used for conducting the field experiment as planting material. The seeds were produced by Sakata Seed Corporation, Japan. The seeds were collected from East Bengal Seed Co. 174, Siddique Bazar, Dhaka-1000, Bangladesh. Green Crown (hybrid) is a medium-maturing, very uniform, large-headed, well-domed broccoli variety with small beads and with excellent texture. The large heads and almost dark green coloration make them very visually attractive. This variety has wide regional adaptability and offers very good uniformity of harvest.

### Preparation of nursery bed and sowing of seeds

Two model seedbeds of size 3 m × 1 m × 0.15 m were prepared at the side of the experimental field. Well-decomposed farm yard manure (FYM) of 15 kg per square meter was applied to the prepared seedbed and mixed with the soil properly. The physical and chemical properties of FYM were analyzed by the soil testing laboratory, Soil Resources Development Institute (SRDI), Jashore, and the details are presented in Supplementary Table S2. The soil of the seedbed was prepared to obtain good tilth to provide a suitable condition for the vigorous growth of young seedlings. Weeds, stubbles, and dried roots of previous crops were removed. Before sowing, Thiram 2.5 g per kg was used to treat the seeds. Seeds were sown on 26 October 2020 during the first year and 13 October 2021 during the second year, respectively in shallow furrows 10–15 cm apart by dropping the seeds at 1–2-cm depth. Regular light watering, hoeing, weeding, plant protection measures, etc. were done from time to time. The seedlings were transplanted 21 days after sowing.

### Transplanting of seedlings

The healthy and 21 days age of uniform seedlings were transplanted to the experimental plots of size 3 m × 2 m, maintaining a spacing of 50 cm × 40 cm as per layout on 16 November 2020 during the first year and on 3 November 2021 during the second year. The transplanting was done in the afternoon, followed by light irrigation.

### Application of chemical fertilizers

The chemical fertilizer of broccoli recommended for the agro-ecological zone (AEZ-11) was used in all the treatments, including the control plot. Based on the soil test, inorganic fertilizers N, P_2_O_5_, K_2_O, and S at the rate of 118, 70, 95, and 2 kg/ha, respectively, were applied in the experimental plots. Urea, triple superphosphate (TSP), muriate of potash (MP), and gypsum were applied as a source of N, P, K, and S, respectively. A full dose of TSP and gypsum were used as basal doses. Urea and MP fertilizers were applied as three equal splits at 15, 30, and 45 days after transplanting as a ring method under moist soil conditions and mixed well with the soil for better utilization (38).

### Intercultural operations

Standard intercultural operations were followed in the entire research plot. Hoeing and weeding were done manually after 30 days of transplanting, followed by a second top dressing with urea and MP fertilizers. The second hoeing, weeding, and earthing up were carried out after 45 days of transplanting, followed by the third top dressing with urea and MP fertilizers. The crop was irrigated immediately after transplanting and then at an interval of 2–3 days until the establishment of seedlings. After this, the crop was irrigated at a regular interval of 7–8 days up to the maturity indices sign. Irrigation was provided with a buried pipe-lined water distribution system. In this system, pumped water is transmitted to the header tank where the pressure head of water is developed. Water then goes to the different points of the crop field through a pipeline laid under the ground surface with the help of outlets. In this system, water can be taken from a lower position to a higher position on the land. Generally, cement concrete (cc) or unplasticized polyvinyl chloride (uPVC) pipes are used to construct underground buried pipe lines. The main considerations for the choice of materials in an irrigation system are the ability of the materials (pipe) to withstand, especially the mechanical stress and temperature to which they may be subjected. Mechanical stress may be due to internal conditions such as water pressure, water acidity, and vacuum (NPI, 2005). To produce safe broccoli, the crop pests and diseases were meticulously managed by daily close observation using biological methods. Completely organic pesticides have been used in the experimental plots. Organic fungicide Dicoprima (750 g ha^−1^) was used to prevent root rot and fusarium wilt of broccoli; 160 g of Dicoprima powder was mixed well with 1 L of water and left aside for at least 6–12 h. Later on, 50 L of water was added to the former solution and thoroughly mixed until homogeneous. The prepared solution was sprayed on the soil 3 days before planting. In the same way, the seedlings in experimental plots were sprayed two more times after the seedlings were planted at 30 days intervals. Under the management of broccoli back diamond, moth insects had been monitored regularly by setting up sex-pheromone using Spodo-O-Lure (Spodoptera Litura Pheromone Lure) (40 number ha^−1^). A yellow sticky board was placed in the experimental plots to control white flies and aphid insects. In addition, Bionim plus (3 mL/L H_2_O) and Neem oil (5 mL/L H_2_O) were sprayed three times at 10–12 day intervals.

### Harvesting

Broccoli heads were harvested in the morning before the soil warms up for the best flavor and when the buds of the head are firm and tight, just before the head's flower. Broccoli heads are cut with sharp scissors or a knife to avoid damaging the stem from the plant, taking at least 15 cm of stem attached to the sprouts. During the first year (2020–2021): five broccoli heads were randomly selected from each replication of all treatments and harvested according to head maturity indices. The first harvesting of broccoli heads was done on 29 January 2020, and the last harvesting was on 3 February 2020 in the first year. Similarly, in the second year first harvesting of broccoli heads was done on 24 January 2021, and the last harvesting on 29 January 2021, respectively. A total of 60 broccoli heads were harvested during the first harvesting, and 40 broccoli heads were harvested during the second harvesting.

### Yield attributing characteristics and head yield

The observation related to head production and its related traits (marketable head weight plant^−1^ (g), diameter and length of heads, and marketable head yield t ha^−1^) was calculated by selecting five plants randomly from each replication of all treatments between 2020 and 2021 and 2021 and 2022. The average was calculated and find out the pooled values for the head's length and diameter, marketable head weight [marketable weight of broccoli means the weight of head and leaves (pruned to at head level)], and marketable head yield.

### Sensory evaluation of color, compactness, and texture

Compactness, sensory evaluation of color, and texture of broccoli were determined in fresh and stored conditions. The sensory evaluation of broccoli was performed using a test panel composed of five trained Sub-Assistant Agriculture Officer (SAAO) panelists. The numerical ratings sensory analyses, such as color, compactness, and texture of stored broccoli were determined and counted from hedonic scales (1 to 5-point) (39) (Table 1), at maximum shelf-life stage both room temperature and cold storage conditions within each level of storage materials separately and the mean values of rating score were calculated and marked the respective color of all treatments. The color, compactness, and texture were determined based on the treatment-wise results from the 2020–2021 and 2021–2022 periods.

**Table 1:** Description of numerical ratings for broccoli quality [According to a 1 to 5-point hedonic scale (39)].

**Scale**	**Ranges of scores**	**Rating for quality attributes of broccoli**		
		**Color**	**Compactness**	**Texture**
1	4.50–5.00	Dark green	Very compact	Highly crispy
2	3.50–4.49	Green	Compact	Crispy
3	2.50–3.49	Light green	Medium compact	Moderately crispy
4	1.50–2.49	Light yellow	Slightly loose	Soft
5	1.00–1.49	Very yellow	Loose	Very soft

### Yellowing (%)

Yellowing was documented visually by cutting the whole green broccoli heads into four parts equally. The yellowing conditions were gradual yellowing and patchy color development.

### Physiological loss in weight (%)

Physiological weight loss (PW) was expressed as % weight loss during storage from the initial weight before storage, and it was documented from the time periodical sampling during storage. The following formula was used for the determination of PLW:


$$\begin{array}{l} \text{PLW}=\frac{\text{Initial weight}-\text{final weight}}{\text{Initial weight}}\times 100 \end{array}$$

### Chemical analysis of fresh and stored broccoli

The treatment-wise samples were analyzed to determine the biochemicals of stored and fresh broccoli in the laboratory. The methods were briefly described chronologically. Carbohydrate content was determined following the AOAC method (40–43).

The following formula was used to determine carbohydrate content:


$$\begin{array}{l} \text{Carbohydrate content }(\%)\text{ = 100 − }(\%\text{ moisture + }\%\text{ ash}\\ \text{+ }\%\text{ protein + }\%\text{ fat + }\%\text{ fiber}) \end{array}$$

Fats of the respective broccoli samples were extracted using the Soxhlet apparatus with n-hexane as a solvent in triplicates (40–43). Approximately 10 g of dry sample of broccoli was extracted for 16 h at 70°C with 40 ml n-hexane using the Soxhlet apparatus. The extract was evaporated to dryness, and the residue was collected. The mean value of each replication under respective treatments was recorded.

The following formula was used to determine fat content:


$$\begin{array}{l} \text{Fat content }\left(\text{\%}\right)=\left\{\left({\text{W}}_{3}-{\text{W}}_{2}\right)/\left({\text{W}}_{2}-{\text{W}}_{1}\right)\right\}\times \;100 \end{array}$$

Where, W_1_ = sample weight (g), W_2_ = extraction thimble weight (g), and W_3_ = extraction thimble weight with the dried crude fat (g).

The protein content was determined following the Micro Kjeldahl procedure (44–48) to determine nitrogen, and then crude protein was calculated by multiplying the nitrogen content by a conversion factor (6.25). The amount of nitrogen present in the sample was converted into ammonium sulfate with sulfuric acid in the presence of a catalyst mixture by digestion at 380°C. The liberated ammonia was distilled with sodium hydroxide solution to absorb by boric acid and then titrated and calculated nitrogen percentage using the following formula.


$$\begin{array}{l} \text{Protein content }(\%)\;=\;[\{({\text{V}}_{1}-{\text{V}}_{2})\times \text{N}\times 14.01\times 100\}\\ \;/(\text{W}\times 1000)]\times \;6.25 \end{array}$$

Where, V_1_ = the amount of sulfuric acid that was used to neutralize the sample, V_2_ = the amount of acid that was used to neutralize the blank, N = normality of the titrant (standard hydrochloric acid, 0.1 N); 14.01 = Molecular weight of nitrogen; 6.25 = total nitrogen to the protein conversion factor.

We pre-incubated the samples using dithiothreitol (DTT) for reducing dehydroascorbic acid to ascorbic acids. Then, ascorbic acids were measured with the reduction of ascorbic acids by 2, 2-dipyridyl (49, 50), which converted Fe^3+^ to Fe^2+^ complexes. The absorbance was taken using a spectrophotometer. Finally, results were expressed as mg/100 g of fresh weight (FW). Total phenol content (TPC) was estimated by the Folin-Ciocalteu reagent (51, 52). A spectrophotometer was used to read the absorbance at 760 nm. TPC was measured using gallic acid standard curves. Finally, results were expressed as mg/100 g FW. The antioxidant activity (AC) (DPPH) was estimated by the radical degradation by DPPH (53, 54). We measured the inhibition % of DPPH equivalent to the control using the equation:


$$\begin{array}{l} \text{AC }\left(\text{\%}\right)=\left(\text{Ab}-\text{AS}/\text{Ab}\right)\times \;100 \end{array}$$

Where, Ab represents the blank sample absorbance and AS is the absorbance of the samples.

Trolox was used as the reference standard, and finally, results were expressed as Trolox equivalent mg/100 g FW.

### Energy calculation

We calculated the energy following the formula of AOAC (55):


$$\begin{array}{l} \text{Energy }(\text{kcal/100 g})\text{ = }(\text{Protein }\times \text{ 4})\text{ + }(\text{Fats }\times \text{ 9})\\ \text{ + }(\text{Carbohydrates }\times \text{ 4}) \end{array}$$

### Shelf-life assessment of broccoli

According to treatment, the average shelf-life (days) was evaluated by taking five samples (452.7 g to 568.78 g for each sample) from each replication using five post-harvest treatments, viz., LDP (35 μm) bag, HDP (15 μm) vacuum pack, 2% eggshell powder solution, 2% ascorbic acid solution, and control condition both at room temperature and cold storage conditions in 2020-2021 and 2021-2022. At the room temperature condition, the storage temperature was 14–22°C with RH 60–65% and at the cold storage, the storage temperature was 4°C with RH 90–95%. The storage room was a one-storied building with a concrete roof and proper ventilation management. Sufficient lighting and ceiling fans were present there. Treatment-wise the broccoli heads were kept in the selective storage facilities and were placed on the table. Concerning cold storage conditions, we used cold storage, Muroley, Razarhat, and Jashore. We meticulously monitored the storage temperature of broccoli heads, both at cold storage and at room temperature conditions. At room temperature conditions, the temperature and relative humidity (RH) were observed by the operation manual for Temperature and Humidity Meter (HTC-2). The fixed temperature of 4°C and RH 90–95% were maintained in the cold storage throughout the storage periods. The shelf-life indicators, viz., deviation of existing color, compactness, texture, percent yellowing, and percent physiological loss in weight (PLW) of broccoli were observed meticulously. Visual and sensory qualities and PLW were observed daily at normal storage and five days intervals at cold storage, and collective results were documented from each replication under selected treatments. The shelf-life (days) had been determined based on the treatment-wise results of the 2020–2021 and 2021–2022 periods.

### Treatments of broccoli

In this study, five post-harvest storage treatments were used, namely: LDP (35 μm) bag, HDP (15 μm) vacuum pack, 2% eggshell powder solution, 2% ascorbic acid solution, and control condition both at room temperature and cold storage conditions during the 2020–2021 and 2021–2022 study periods (Supplementary Figure S1). In the case of the LDP (35 μm) bag, the single sample of each replication was kept inside the package carefully and air-tied the bag as soon as possible. Similarly, in the case of the HDP (15 μm) vacuum pack, the single sample of each replication was kept inside the package carefully and locked in the bag as soon as possible. The dip treatment in 2% eggshell powder solution for 5 min to increase the shelf-life of broccoli heads was investigated. Similarly, the dip treatment in 2% ascorbic acid solution for 5 min to increase the shelf-life of broccoli heads was also investigated.

### Determination of CO_2_ and O_2_ conditions and respiration rate inside the packages

We measured CO_2_ and O_2_ conditions inside the packages using the Q_2_-Portable O_2_/CO_2_ analyzer (Shenzhen Empaer Technology Co. Ltd., Shenzhen, China). We also measured the respiration rate inside the packages using 3051H Fruit and Vegetable Breathing Tester (Zhejiang Top Cloud-Agri Technology Co. Ltd. China).

### Economic performance

The cost of cultivation per hectare under different treatments was found based on expenditure incurred on different operations production of the crop separately under each treatment of both seasons. Gross return was found based on the market price of the product at the time when the product was ready for sale. The treatment-wise net return per hectare was found by deducting the cost of cultivation from the gross return per hectare. The benefit–cost ratio (BCR) for each treatment under investigation was calculated based on the present market prices of inputs and outputs to find out the maximum profitable treatment. The benefit–cost ratio (BCR) was found as follows:


$$\begin{array}{l} \text{Benefit Cost Ratio }\left(\text{BCR}\right)=\frac{\text{Gross returns}}{\text{Cost of cultivation}} \end{array}$$

### Statistical analysis

The raw data were compiled by taking the means of all the plants taken for each treatment and replication for different traits. Then, mean data from both years (2020–2021 and 2021–2022) were pooled before statistical analysis. The recorded pooled data were analyzed using the Statistical Tool for Agricultural Research (STAR) program, and the pooled values were tested for significance using Tukey's test at 5 or 1% levels.

## Results and discussions

### Yield attributing characteristics and head yield

**Figure 1:**
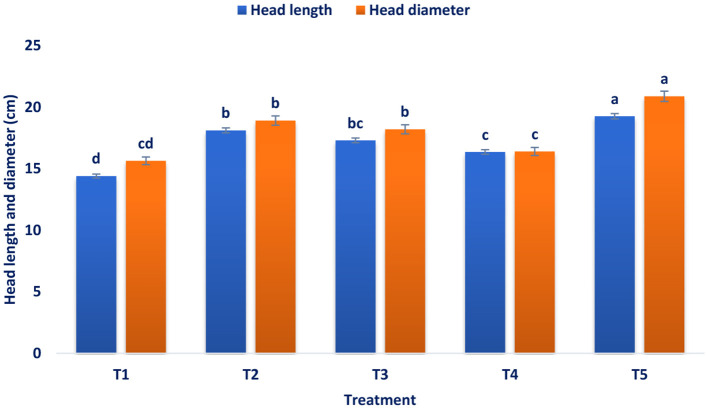
Influence of pre-harvest foliar application of mineral nutrients on the head length and diameter of broccoli at the marketable stage. T_1_ = control, T_2_ = B, T_3_ = Zn, T_4_ = Mo, and T_5_ = B + Zn + Mo, 0.40%, respectively.

**Figure 2:**
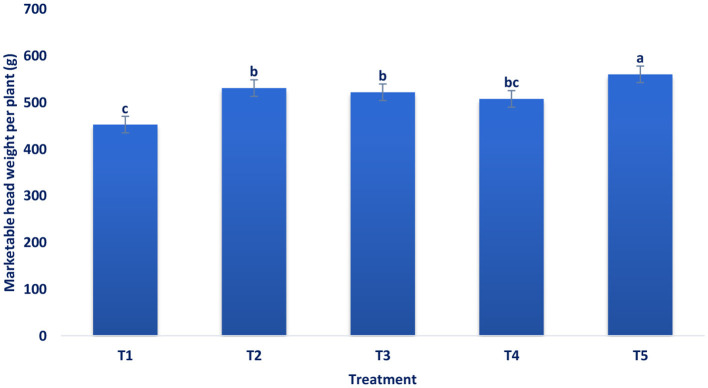
Influence of pre-harvest foliar application of mineral nutrients on marketable head weight plant^−1^ (g) of broccoli at the marketable stage. T_1_ = control, T_2_ = B, T_3_ = Zn, T_4_ = Mo, and T_5_ = B + Zn + Mo, 0.40%, respectively.

**Figure 3:**
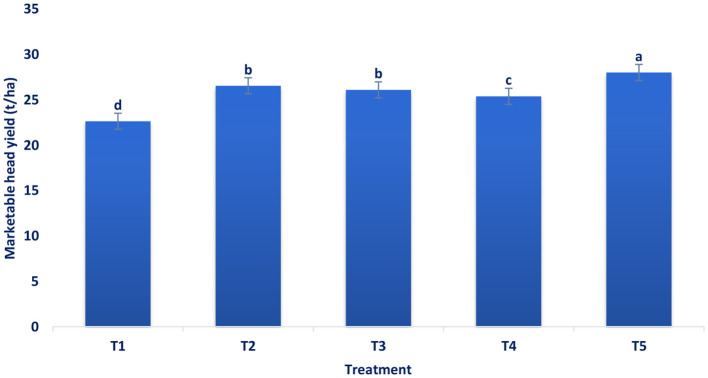
Influence of pre-harvest foliar application of mineral nutrients on the marketable head yield t ha^−1^ of broccoli. T_1_ = control, T_2_ = B, T_3_ = Zn, T_4_ = Mo, and T_5_ = B + Zn + Mo, 0.40%, respectively.

**Figure 4:**
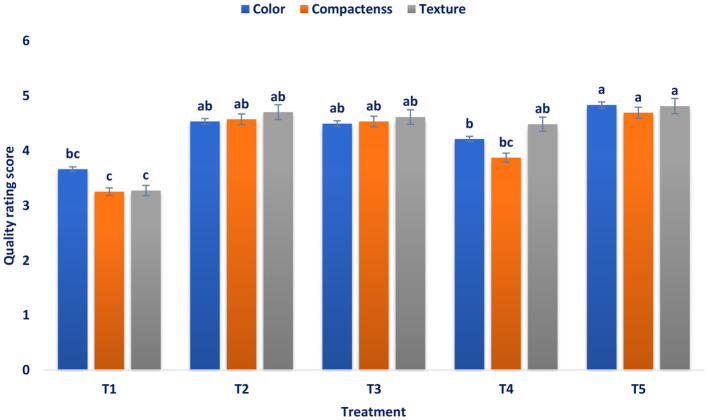
Influence of pre-harvest foliar application of mineral nutrients on quality indices of fresh broccoli. T_1_ = control, T_1_ = control, T_2_ = B, T_3_ = Zn, T_4_ = Mo, and T_5_ = B + Zn + Mo, 0.40%, respectively.

Figures 1–4 revealed that foliar spray of mineral significantly (*p* ≤ 0.05) increased the head length and diameter, marketable head weight, and marketable head yield of broccoli. Among the treatments, the highest head length (19.25 cm) and head diameter (20.87 cm) were documented in B + Zn + Mo, followed by B with a head length of 18.44 cm, head diameter of 18.9 cm and Zn with a head length of 17.29 cm and head diameter of 18.39 cm (Figure 1). At a 5% level of significance, the maximum pooled value of marketable head weight plant^−1^ (560.33 g) was recorded in B + Zn + Mo followed by B (530.94 g) and Zn (521.92 g) (Figure 2). There was no statistical difference between B and Zn treated plots for marketable head weight plant^−1^. The maximum pooled value of marketable head yield (28.02 t ha^−1^) was recorded in B + Zn + Mo followed by B (26.55 t ha^−1^) and Zn (26.1 t ha^−1^) (Figure 3). The marketable head yield had no statistical difference between B and Zn treated plots. Whereas, in the control treatment, the minimum head length, head diameter, marketable head weight plant^−1^, and marketable head yield were 14.39 cm, 15.63 cm, 452.7 g, and 22.64 t ha^−1^, respectively.

Maximum head length and diameter were obtained from the spraying of combined B, Zn, and Mo. The supply of sufficient essential elements may be the reason for more vegetative growth. The results were corroborative of the results of Singh et al. (56) in broccoli and Bairwa et al. (57) in cauliflower. The synergistic action of these elements increased carbohydrate accumulation and photosynthesis rate in the head and enhanced diameter and length. Chowdhury and Sikder (34) found similar increments in the diameter and length of broccoli. The application of treatment B + Zn + Mo increased the marketable head yield by 19.20% compared to the control. Physiological activities in broccoli, viz., protein, and chlorophyll synthesis, cell elongation and division, carbohydrate metabolism, amino acid formation, sugar translocation, and various enzymatic reactions, were accelerating due to foliar spay of Zn, B, and Mo, which leads to more carbohydrate accumulation in broccoli heads. This might be due to the combined foliar application of the advanced and subsequently increased the broccoli yield. These results conform with the results on broccoli (34). Singh et al. (56) observed that the application of B + Mn + Zn produced a significant maximum yield contributing traits like head diameter, length, and marketable head yield of broccoli. In broccoli, application from zero to 1.5 kg/ha B significantly enhanced leaves per plant, plant height, leaf length and width, plant spread, main head weight, and head yield (24). Xaxa et al. (58) showed that the head yield of broccoli was significantly influenced by the different treatment combinations of micronutrients. Patel et al. (25) observed that the combined fertilization of B (2.5 kg/ha), Mo (0.5 kg/ha), manganese (3 kg/ha), and Zn (2 kg/ha) had the highest head yield. Patel et al. (25) also investigated that compared to RDF, the combined application of B and Mo was more effective than their sole application. Application of RDF + borax (1.5%) + ammonium molybdate (2.5%) was found as the best combination for the maximum head yield of broccoli. Similar findings were also recorded by Bairwa et al. (57) in cauliflower, who showed that application of 2 kg ha^−1^ ammonium molybdate + 25 kg ha^−1^ ZnSO_4_ + RDF (100%) and 20 kg ha^−1^ borax displayed most substantial effect on yield and yield attributing characteristics of cauliflower compared to control (100% RDF). It revealed from the current study that different treatments spray increased the head yield, and combined micronutrients B, Mo, and Zn demonstrated the highest values. Micronutrient spray promotes food material production, translocation, transformation, and distribution from leaves to the sink tissue in the broccoli heads. The higher head yield might be attributed due to the application of that mixture of micronutrients contributed to head yield because of the improved availability of vital elements at optimum stages of growth, which ultimately promotes the rate of metabolism in plants (25). Augmented activities of metabolic caused greater assimilation of carbohydrates and proteins that additionally led to greater nutrient uptake and yields. Furthermore, micronutrients spray accumulated more photosynthates and increased other metabolic activity for the biosynthesis of numerous primary and secondary metabolites for cell elongation and division, which improved head yield and its related characters (59). Mo promotes plant weight and leaf characteristics and may be a noteworthy consequence of the augmentation of head yield and its related characteristics. Moreover, Mo spray stimulates the augmentation of the metabolic pools essential for the amalgamation of saccharides, in addition to enhancing the capacity of photosynthetic (60). Phosphorous and B in the plant resulted from the synergistic interaction effect of greater head yield (61). Zn and the P application may create the synergetic effect that serves as an energy source for auxin synthesis. Moniruzzaman et al. (24) reported enhanced photosynthetic reactions in broccoli with the application of Zn and B.

### Physico-chemical analysis of fresh broccoli

#### Sensory evaluation of color, compactness, and texture

The dark green color and very compact and highly crispy broccoli heads are the most important quality attributes which determine its marketability and market value. From the experimental results shown in Figure 4, it was found that pre-harvest foliar application of B, Zn, B + Zn + Mo, and significantly (*p* ≤ 0.01) increased the quality attributes, viz., texture, compactness, and color of broccoli. According to the 1–5-point hedonic scale of Ranganna (39), the maximum pooled value of texture (4.81), compactness (4.69), and color (4.83) were determined in B + Zn + Mo followed by B with compactness (4.57), texture (4.70), and color (4.53) and Zn with the compactness (4.53), color (4.49), and texture (4.61). Whereas, the minimum compactness (3.25), color (3.66), and texture (3.27) were observed in control. It may be owing to the effects of the foliar spray of B, Zn, and Mo that influenced the length of the duration of the post-harvest life of broccoli through increased nutrient uptake by the plants during the growing period. It also developed water-conducting tissue for maintaining the quality attributes, including compactness, color, and texture of broccoli, which is in accordance with the previous study of Mohamed et al. (62) in broccoli. The application of foliar mineral nutrients, particularly B, Zn, and Mo, has a beneficial role in improving the quality of broccoli due to their involvement as a catalyst of various enzymes and other physiologically active molecules (63). Pre-harvest application of Zn plays an important role in the photosynthesis process (64), which increases color density. Zn increases the net photosynthetic rate, and chlorophyll content is probably responsible for the green color of broccoli heads (65). Mo presumably maintains cell firmness and its structure of walls and helps in retarding weight loss of broccoli (66). Duffy (67) stated that Zn improved turgidity and efficiency of water use in tissues and helps to maintain greater moisture levels inside the tissues of broccoli heads. B increases the synthesis of secondary metabolites for the biosynthesis of lignin (68). Thus, a rise in lignin as a result of spraying higher doses of B in broccoli tissue may minimize water loss.

### Chemical analysis of fresh broccoli heads

#### Protein

From Table 2, it was found that foliar spray of nutrients significantly (*p* ≤ 0.01) augmented protein content in broccoli heads. Among the treatments, the maximum protein (3.55%) was recorded in Mo, followed by B + Zn + Mo (3.33%) and Zn (3.31%). Whereas, the minimum protein (2.5%) was observed in the control. Mo may involve in the absorption of nitrogen and nitrogen metabolism, which leads to higher protein content in broccoli heads (69). B plays a pivotal role in nitrogen (N) metabolism as it enhances nitrate levels and reduces nitrate reductase activity under limited B conditions (70, 71).

**Table 2:** Pre-harvest foliar application effect of mineral nutrient sources on nutrient content in fresh broccoli heads.

**Treatment**	**Nutrient contents in fresh broccoli head**						
	**Protein (%)**	**Fat (%)**	**Carbohydrates (%)**	**Energy (kcal/100 g)**	**Vitamin C (mg/100 g)**	**Antioxidants (mg/100 g)**	**Phenols (mg/100 g)**
T_1_	2.50 ± 0.01c	0.3821 ± 0.02c	2.82 ± 0.2c	24.72 ± 0.1d	70.3 ± 0.02d	55.71 ± 0.1d	26.96 ± 0.1d
T_2_	2.86 ± 0.3b	0.4331 ± 0.1ab	4.18 ± 0.3bc	32.03 ± 0.2bc	84.95 ± 0.03ab	68.45 ± 0.3b	38.42 ± 0.3b
T_3_	3.31 ± 0.03abc	0.4362 ± 0.01a	4.47 ± 0.1ab	35.25 ± 0.4b	85.99 ± 0.4ab	70.62 ± 0.04ab	40.24 ± 0.05ab
T_4_	3.55 ± 0.04a	0.4155 ± 0.3b	3.43 ± 0.1b	31.64 ± 0.01c	81.31 ± 0.04abc	65.13 ± 0.1bc	34.71 ± 0.02bc
T_5_	3.33 ± 0.5ab	0.4375 ± 0.01a	5.25 ± 0.2a	38.26 ± 0.1a	87.75 ± 0.01a	72.45 ± 0.2a	41.03 ± 0.01a
Level of Significance	^**^	^**^	^**^	^**^	^**^	^**^	^**^

T_1_ = control, T_2_ = B, T_3_ = Zn, T_4_ = Mo, and T_5_ = B + Zn + Mo, 0.40%, respectively. ^**^Significant level at 1% level of significance, in a column means having a similar letter(s) are not significantly different.

#### Fat

It was revealed from Table 2 that foliar spray of nutrients increased the fat content significantly (*p* ≤ 0.01) in broccoli heads. Among the treatments, the maximum fat (0.4375%) was recorded in B + Zn + Mo and Zn (0.4362%) and followed by B (0.4331%). Whereas, the minimum fats of 0.3821% were noted in the control.

#### Carbohydrates

According to experimental results in Table 2, it was found that foliar spray of nutrients significantly (*p* ≤ 0.01) increased the carbohydrate content in broccoli heads. Among the treatments, the maximum carbohydrates (5.25%) were recorded in B + Zn + Mo followed by Zn (4.47%) and B (4.18%), which were statistically similar. Whereas, the minimum carbohydrates (2.82%) were noted in the control. It is owing to greater vegetative growth and metabolic activities for accumulation of more carbohydrates in broccoli heads deferential as a result of nutrient spray. Sharma (33) and Singh et al. (28) also found the results in broccoli to support our present findings. The literature has shown that foliar spray of Zn during pre-harvest displayed the highest carbohydrate; however, Mo increased the reducing sugar (33).

#### Energy

Table 2 revealed that the foliar spray of nutrients displayed significant (*p* ≤ 0.01) effects on the energy produced in the broccoli heads. Among the treatments, the maximum energy (38.26 kcal/100 g) was recorded in B + Zn + Mo, followed by Zn (35.25 kcal/100 g) and B (32.03 kcal/100 g). Whereas, the minimum energy (24.72 kcal/100 g) was recorded in the control. This might be due to the accumulation of fats in the broccoli heads from the combined foliar application of mineral nutrients. Beta-oxidation of fats produces more adenosine triphosphate (ATP) compared to carbohydrates and proteins. These high energy-rich compounds ATPs release energy in the broccoli heads by breaking into ADP and inorganic phosphate. We know that energy is released from fat, protein, and carbohydrate. In our study, a combined spray of B + Zn + Mo increased fat, protein, and carbohydrate content in broccoli heads, and the increment of these compounds may result in the augmentation of energy heads.

#### Vitamin C

Vitamin C content differed significantly (*p* ≤ 0.01) in the broccoli heads with the application of nutrients in foliage (Table 2). Among the treatments, the maximum vitamin C (87.75 mg/100 g) was recorded in B + Zn + Mo followed by Zn (85.99 mg/100 g) and B (84.95 mg/100 g). Whereas, the minimum vitamin C was observed (70.3 mg/100 g) in the control. B, Zn, and Mo meet up need-based essential nutrients to plants and may create the synergistic effects to enhance the photosynthetic rate for greater development and growth of broccoli which finally mobilize the ascorbic acid biosynthesis to accumulate vitamin C in broccoli heads. These results are consistent with the results of broccoli (25, 34, 56).

#### Antioxidants

The application of foliar nutrients significantly (*p* ≤ 0.01) influenced the antioxidant content in broccoli heads (Table 2). Among the treatments, the maximum antioxidants (72.45 mg/100 g) were observed in B + Zn + Mo followed by Zn (70.62 mg/100 g) and B (68.45 mg/100 g), which were statistically similar. The minimum antioxidants (55.71 mg/100 g) were recorded in the control. The previous literature (72) has shown that application of minerals stimulates polyphenolic biosynthesis and antioxidant activity in plants. As high polyphenols content highly correlates with high antioxidant potential (73, 74), broccoli heads showed high antioxidant potential.

#### Total phenol content

Nutrient spray in foliage responded significantly (*p* ≤ 0.01) to the total phenol content in broccoli heads (Table 2). Among the treatments, the maximum phenols (41.03 mg/100 g) were observed in B + Zn + Mo, followed by Zn (40.24 mg/100 g) and B (38.42 mg/100 g), which were statistically similar. Whereas, the minimum phenol (26.96 mg/100 g) was noted in the control. The antioxidant activity of phenols is mainly due to their redox properties, which allow them to act as antioxidants (75). Phenolics have numerous groups of simple phenols and phenolic acids, including hydroxybenzoic acids and hydroxycinnamic acids (76, 77). Flavonoids such as flavonols, flavanols, flavones, flavanones, isoflavones, and anthocyanins (78), which detoxify the reactive oxygen species (ROS) (79, 80), protect against many degenerative diseases like cancer and cardiovascular disorders (9). The previous literature (72) has shown that application of minerals stimulates polyphenolic biosynthesis in plants which correlates to our current results. Glucosinolates are abundant phenolic compounds in broccoli. Foliar zinc application significantly increased the sulforaphane content in broccoli heads (81). Sulfur and boron application increased glucosinolate concentration in rapeseeds (82). From the literature, we found that phenolic compounds in broccoli were increased, which supports the augmentation of total polyphenols in our current study.

### Shelf-life

Shelf-life is always dependent on the type of vegetables and the conditions in which they are stored. At room temperature conditions (14–22°C with RH 60–65%), the samples were stored for 1.75–7.25 days, LDP (35 μm) bag packaging samples were stored for 3.29–5.53 days, HDP (15 μm) vacuum packaging samples were stored for 3.85–7.25 days, 2% eggshell powder solution-treated broccoli heads were stored for 2.33–3.73 days, 2% ascorbic acid solution-treated broccoli heads samples were stored for 2.25–3.69 days, and control conditioned broccoli heads were stored for 1.75–3.25 days. In the cold storage (RH 90–95% and 4°C), the samples were stored for 11.04–24.73 days. LDP (35 μm) bag packaging samples were stored for 15.04 to 22.52 days, HDP (15 μm) vacuum packaging samples were stored for 16.89–24.73 days, 2% eggshell powder solution-treated broccoli heads were stored for 12.39–15.37 days, 2% ascorbic acid solution-treated broccoli heads samples were stored for 12.31 to 15.25 days, and control conditioned broccoli heads were stored for 11.04–13.35 days. Evident from the experimental results revealed that foliar spray of minerals and post-harvest packaging and chemical treatments significantly (*p* ≤ 0.01) influenced the shelf-life of broccoli (Table 3). At a 1% level of significance, the treatment combination of T_5_ × HDP confirmed a maximum shelf-life of 7.05 days at room temperature (RH 60–65% and 14–22°C) which was statistically different from others. The treatment combination of T_2_ × HDP gave the second maximum shelf-life of 6.13 days at room temperature, followed by T_3_ × HDP with 5.75 days and T_5_ × LDP with 5.49 days which were statistically different. While the treatment combination of T_1_ × con. showed a minimum shelf-life of 1.75 days at room temperature conditions. Similarly, at a 1% level of significance, the treatment combination of T_5_ × HDP confirmed a maximum shelf-life of 24.55 days at cold storage (90–95% RH and 4°C) conditions which were statistically different from others. The treatment combination of T_2_ × HDP gave the second maximum shelf-life of 23.35 days at cold storage, followed by T_3_ × HDP with 22.69 days, T_5_ × LDP with 21.75 days, T_2_ × LDP with 21.36 days, and T_3_ × LDP with 20.53 days, which were statistically similar. While the treatment combination of T_1_ × con. showed a minimum shelf-life of 11.04 days under cold storage conditions. At the temperature of 14°C, the oxygen and carbon dioxide conditions inside the LDP packages were 45% O_2_ + 55% CO_2_, and inside the HDP packages were 48% O_2_ + 52% CO_2_ (Table 4). At the temperature of 22°C, the oxygen and carbon dioxide conditions inside the LDP packages were 33% O_2_ + 67% CO_2_, and inside the HDP packages were 36% O_2_ + 64% CO_2_. On the other hand, in cold storage, the O_2_ and CO_2_ conditions inside the LDP packages were 60% O_2_ + 40% CO_2_, and inside the HDP packages, were 65% O_2_ + 35% CO_2_ (Table 4). Similarly, at the temperature of 14°C, the respiration rate inside the LDP packages was 80–90 mg CO_2_ kg^−1^ h^−1^; inside the HDP packages was 78–80 mg CO_2_ kg^−1^ h^−1^. At the temperature of 22°C, the respiration rate inside the LDP packages was 204–307 mg CO_2_ kg^−1^ h^−1^, and inside the HDP packages was 190–255 mg CO_2_ kg^−1^ h^−1^. On the other hand, in cold storage, the respiration rate inside the LDP packages was 15–20, and inside the HDP packages was 10–11 mg CO_2_ kg^−1^ h^−1^ (Table 4). The treatment combination of T_5_ × HDP significantly gave the maximum shelf-life both at room temperature (RH 60–65% and 14–22°C) and in cold storage (RH 90–95% and 4°C). Foliar application of B, Zn, and Mo might be improved nutrients uptake and greater development of water-conducting tissue in plants during the growth period and ultimately increased the length of the duration of post-harvest life, compactness, color, and texture of broccoli by the plants. These findings are supported by Mohamed et al. (62) in broccoli. A previous study showed that polyethylene bags (HDP) enhance the efficiency of the post-harvest shelf-life of broccoli which is corroborative to the current study (29, 35). The application of foliar mineral nutrients, particularly B, Zn, and Mo has a beneficial role in improving the quality of broccoli due to their involvement as a catalyst of various enzymes and other physiologically active molecules (62). Foliar minerals minimized the respiration rate, strengthened the cell wall, and protected available moisture in broccoli heads which prevented yellowing and weight loss and subsequently preserved shelf-life during storage (83). The majority of the interaction effects of post-harvest treatments, such as eggshells (2%) and ascorbic acids (2%) and pre-harvest nutrients sprays like Mo, B, Zn, and Mo + B + Zn demonstrated medium to low shelf-life. Some researchers also dipped fruit in ascorbic acid and eggshell powder solutions and found suitable post-harvest treatments for extending shelf-life and increasing the keeping quality intact for a long-time quality of fresh vegetables and fruits (36, 37).

**Table 3:** Influence of pre-harvest foliar application of mineral nutrients and post-harvest packaging and chemical treatments on shelf-life of broccoli under different storage temperatures.

**Treatment combination**	**Shelf-life (days)**	
	**At room temperature condition (14-22**°**C with RH 60-65%)**	**In cold storage condition (4**°**C with RH 90-95%)**
T_1_ × LDP	3.29 ± 0.02gh	15.04 ± 0.01cd
T_2_ × LDP	5.33 ± 0.03de	21.36 ± 0.01b
T_3_ × LDP	4.85 ± 0.05e	20.53 ± 0.04bc
T_4_ × LDP	4.37 ± 0.01f	18.12 ± 0.05bc
T_5_ × LDP	5.49 ± 0.01d	21.75 ± 0.03b
T_1_ × HDP	3.85 ± 0.05e	16.89 ± 0.1c
T_2_ × HDP	6.13 ± 0.04b	23.35 ± 0.02b
T_3_ × HDP	5.75 ± 0.01c	22.69 ± 0.04b
T_4_ × HDP	5.23 ± 0.03de	21.65 ± 0.03b
T_5_ × HDP	7.05 ± 0.01a	24.55 ± 0.1a
T_1_ × ESP	2.33 ± 0.03ij	12.39 ± 0.05de
T_2_ × ESP	3.45 ± 0.01g	14.52 ± 0.02cd
T_3_ × ESP	3.25 ± 0.02gh	14.25 ± 0.1cd
T_4_ × ESP	2.75 ± 0.01hi	13.74 ± 0.03d
T_5_ × ESP	3.69 ± 0.01e	15.25 ± 0.04c
T_1_× AA	2.25 ± 0.02ij	12.31 ± 0.01de
T_2_ × AA	3.33 ± 0.04gh	14.11 ± 0.05cd
T_3_ × AA	3.13 ± 0.03hi	13.94 ± 0.02cd
T_4_ × AA	2.45 ± 0.01i	13.31 ± 0.03de
T_5_ × AA	3.63 ± 0.05e	15.05 ± 0.04c
T_1_ × Con	1.75 ± 0.04j	11.04 ± 0.01f
T_2_ × Con	3.05 ± 0.02hi	12.55 ± 0.02de
T_3_ × Con	2.55 ± 0.05i	12.33 ± 0.04de
T_4_× Con	1.93 ± 0.02ij	12.04 ± 0.05e
T_5_ × Con	3.15 ± 0.01gh	13.25 ± 0.03de
Level of significance	^**^	^**^

T_1_ = control, T_2_ = B, T_3_ = Zn, T_4_ = Mo, and T_5_ = B + Zn + Mo, 0.40%, respectively. LDP = low-density polyethylene (35 μm) bag, HDP = high-density polyethylene (15 μm) vacuum pack, ESP = 2% eggshell powder solution, AA = 2% ascorbic acid solution, and Con = control condition. ^**^Significant level at 1%, level of significance, in a column, means having a similar letter(s) are not significantly different.

**Table 4:** CO_2_ and O_2_ conditions and respiration rate inside the packages.

**Parameters**	**Temperature**					
	**In cold storage condition**		**At room temperature condition**			
	**4**°**C**		**14**°**C**		**22**°**C**	
	**LDP**	**HDP**	**LDP**	**HDP**	**LDP**	**HDP**
Respiration rate (mg CO_2_ kg^−1^ h^−1^)	15–20	10–11	80–90	78–80	204–307	190–255
Oxygen condition (%)	60	65	45	48	33	36
Carbon dioxide condition (%)	40	35	55	52	67	64

LDP, low-density polyethylene; HDL, high-density polyethylene.

The reason for the highest shelf-life in the HDP vacuum pack (15 μm) may be its sophisticated techniques which delayed the physiological deterioration of broccoli heads. In HDP vacuum (15 μm) packaging, control gas exchange around air and CO_2_ and O_2_ levels decelerated the alteration of starch to sugars. Cold storage prevented decaying, rotting, and chilling injury in broccoli heads and maintained a more green color. Furthermore, low temperatures during storage reduced respiration rate and delayed the senescence of broccoli heads. A foliar spray of nutrients in broccoli and a storage HDP vacuum pack (15 μm) protected the degradation of chlorophyll and production of ethylene. This combination might control the yellowing of broccoli. These results of shelf-life corroborated the results of broccoli (32).

This study also observed that broccoli heads, when kept in control (without using storage materials) at room temperature conditions, the shelf-life of broccoli deteriorated because of the higher respiratory rate, moisture loss, and physiological changes that occurred very faster rate compared to other post-harvest storage treatments. These findings are in accordance with the previous findings of Ferdousi et al. (83) and Roni et al. (84) on broccoli.

### Physico-chemicals at maximum shelf-life of storage broccoli

#### Sensory evaluation of color, compactness, and texture

Changes in color, compactness, and texture values are important factors affecting visual qualities during the post-storage period (Table 5). Foliar spray of nutrients and packaging and chemical treatments during storage significantly (*p* ≤ 0.01) influenced the color, compactness, and texture of broccoli. Among the treatments, the maximum color (rating 3.75), compactness (rating 3.81), and texture (rating 3.71) were documented in T_5_ × HDP at cold storage (RH 90–95% and 4°C) after 24 days of storage periods. Whereas, the minimum color (rating 1.94), compactness (rating 2.08), and texture (rating 1.95) were observed in T_1_ × con. under cold storage after 11 days. Similarly, storage broccoli heads at room temperature (RH 60–65% and 14–22°C) demonstrated the maximum color (3.65), compactness (3.73), and texture (3.66) in the treatment combination of T_5_ × HDP after 7 days. Whereas, the minimum color (1.89), compactness (2.17), and texture (1.42) were observed in T_1_ × con. at room temperature (RH 60–65% and 14–22°C) condition after 2 days. All the interaction effects of post-harvest treatments [eggshells (2%) and ascorbic acids (2%)] and pre-harvest nutrients spray (Mo, B, Zn, and Mo + B + Zn) resulted in medium to low color, compactness, and texture. In the previous study, some workers also dipped fruit in ascorbic acid and eggshell powder solutions and found suitable post-harvest treatments to preserve the quality of fresh vegetables and fruits (36, 37).

**Table 5:** Effects of pre-harvest foliar application of mineral nutrient sources and post-harvest packaging and chemical treatments on quality attributes in storage broccoli heads under different storage temperatures at the maximum shelf-life stage.

**Treatment combination**	**Color**		**Compactness**		**Texture**	

	**At room temperature condition (14-22**°**C)**	**In cold storage condition (4**°**C)**	**At room temperature condition (14-22**°**C)**	**In cold storage condition (4**°**C)**	**At room temperature condition (14-22**°**C)**	**In cold storage condition (4**°**C)**
T_1_ × LDP	2.31 ± 0.2c	2.27 ± 0.03d	2.40 ± 0.09c	2.38 ± 0.4c	2.46 ± 0.08c	2.31 ± 0.02c
T_2_ × LDP	3.43 ± 0.5ab	3.30 ± 0.07ab	3.68 ± 0.04a	3.70 ± 0.01ab	3.36 ± 0.04ab	3.21 ± 0.01ab
T_3_ × LDP	3.27 ± 0.1ab	3.15 ± 0.04abc	3.42 ± 0.02ab	3.47 ± 0.07ab	3.16 ± 0.01abc	3.15 ± 0.03ab
T_4_ × LDP	3.10 ± 0.3ab	3.16 ± 0.01ab	3.18 ± 0.1abc	3.27 ± 0.01ab	2.93 ± 0.03abc	2.90 ± 0.02abc
T_5_ × LDP	3.57 ± 0.4a	3.71 ± 0.06a	3.75 ± 0.03a	3.76 ± 0.06a	3.55 ± 0.06a	3.67 ± 0.01a
T_1_ × HDP	2.39 ± 0.01c	2.43 ± 0.01c	2.46 ± 0.02c	2.42 ± 0.09c	2.48 ± 0.02c	2.38 ± 0.08c
T_2_ × HDP	3.57 ± 0.5ab	3.41 ± 0.6ab	3.70 ± 0.01a	3.85 ± 0.07a	3.43 ± 0.08ab	3.25 ± 0.01ab
T_3_ × HDP	3.35 ± 0.4ab	3.21 ± 0.2abc	3.56 ± 0.08ab	3.68 ± 0.03a	3.26 ± 0.03ab	3.29 ± 0.05ab
T_4_ × HDP	3.19 ± 0.02bc	3.25 ± 0.01ab	3.29 ± 0.07ab	3.52 ± 0.02ab	3.11 ± 0.07abc	3.16 ± 0.08ab
T_5_ × HDP	3.65 ± 0.7a	3.75 ± 0.07a	3.73 ± 0.5a	3.81 ± 0.01a	3.66 ± 0.8a	3.71 ± 0.08a
T_1_ × ESP	2.07 ± 0.9d	2.11 ± 0.01c	2.37 ± 0.4c	2.17 ± 0.07d	2.31 ± 0.6c	2.27 ± 0.08c
T_2_ × ESP	3.29 ± 0.6ab	3.21 ± 0.06a	3.42 ± 0.8a	3.45 ± 0.01ab	3.23 ± 0.07abc	3.10 ± 0.09ab
T_3_ × ESP	3.05±abc	3.07 ± 0.03ab	3.21 ± 0.6ab	3.22 ± 0.08ab	3.08 ± 0.01abc	3.02 ± 0.01abc
T_4_ × ESP	2.89 ± 0.04abc	3.05 ± 0.04ab	2.91 ± 0.04abc	3.09 ± 0.07abc	2.72 ± 0.07abc	2.62 ± 0.01bc
T_5_ × ESP	3.51 ± 0.8a	3.57 ± 0.02a	3.45 ± 0.06a	3.69 ± 0.03a	3.43 ± 0.09a	3.591 ± 0.5a
T_1_× AA	2.01 ± 0.01d	2.05 ± 0.04d	2.34 ± 0.07c	2.08 ± 0.2d	2.27 ± 0.8c	1.95 ± 0.7c
T_2_ × AA	3.30 ± 0.09ab	3.07 ± 0.09ab	3.40 ± 0.01a	3.37 ± 0.01ab	3.28 ± 0.02ab	3.04 ± 0.05ab
T_3_ × AA	2.95 ± 0.07abc	2.85 ± 0.03abc	3.17 ± 0.1ab	3.10 ± 0.5ab	3.03 ± 0.06abc	2.90 ± 0.4ab
T_4_ × AA	12.80 ± 0.05abc	2.91 ± 0.06ab	2.87 ± 0.3bc	2.89 ± 0.4bc	2.75 ± 0.02abc	2.59 ± 0.1bc
T_5_ × AA	3.45 ± 0.02a	3.47 ± 0.01a	3.41 ± 0.08a	3.63 ± 0.3a	3.36 ± 0.7a	3.45 ± 0.4a
T_1_ × Con	1.72 ± 0.08c	1.94 ± 0.02d	2.17 ± 0.01c	2.08 ± 0.2d	1.42 ± 0.09c	1.95 ± 0.3c
T_2_ × Con	2.6 ± 0.06ab	3.08 ± 0.07ab	3.38 ± 0.07a	3.38 ± 0.4ab	2.89 ± 0.07ab	2.99 ± 0.1ab
T_3_ × Con	2.37 ± 0.03abc	2.77 ± 0.01abc	3.09 ± 0.3ab	3.09 ± 0.4ab	2.72 ± 0.01ab	2.89 ± 0.3ab
T_4_× Con	2.18 ± 0.7abc	2.83 ± 0.02ab	2.66 ± 0.04abc	2.81 ± 0.7bc	2.54 ± 0.9ab	2.53 ± 0.4bc
T_5_ × Con	2.81 ± 0.04a	3.45 ± 0.01a	3.36 ± 0.01a	3.65 ± 0.2a	3.25 ± 0.1a	3.41 ± 0.3a
Level of significance	**	**	**	**	**	**

T_1_ = control, T_2_ = B, T_3_ = Zn, T_4_ = Mo, and T_5_ = B + Zn + Mo, 0.40%, respectively. LDP = Low-density polyethylene (35 μm) bag, HDP = High-density polyethylene (15 μm) vacuum pack, ESP = 2% eggshell powder solution, AA = 2% ascorbic acid solution, and Con = control condition. ^**^Significant level at 1%, level of significance, in a column, means having a similar letter(s) are not significantly different.

Among the treatment combinations, the maximum green color was retained intact in T_5_ × HDP at the end of the storage period at both room temperature and cold storage conditions. The yellowing was more in the broccoli without any packaging or treatment. It was evident that the minimum yellow color of broccoli was recorded in both the storage conditions using an HDP vacuum pack (15 μm) might be due to its sophisticated techniques, which delayed and protected the physiological deterioration of the broccoli heads. In the HDP vacuum pack (15 μm), gas exchange was more controlled in the surrounding air, and CO_2_ and O_2_ levels were declined to stored broccoli heads that ultimately converted the starch to sugars. Moreover, low-temperature storage of broccoli heads reduced the respiration rate and delayed senescence. This might have restricted the yellowing of broccoli. The findings of the present investigation corroborate the findings of Chingtham and Banik (29) on broccoli. These results agreed with the results of De Beer and Crouch (35), who displayed a greater yellowing % from 7 days of storage at 0 °C. Exactly 25% yellowing was found in control-treated broccoli heads after 21 days of storage which had reached unsaleable. Packed broccoli confirmed color scores of <15–20% for long storage. The increasing trend of the yellowing of broccoli during the study period was also supported by Eason et al. (85), who detected that control condition broccoli heads beginning the senesce and yellowing after 2 days. They also observed that the senescence of broccoli heads was delayed with a shelf-life of 30 and 24 days when it was stored at 0°C and 5°C, respectively.

Maximum compactness and texture scores decreased with the advancement of the period of storage. These findings conform with that of Chingtham and Banik (29) in broccoli. Beer and Crouch (35) showed that HDP (15 μm) bags, in terms of yellowing percentage, extended storability by more than 14 days, compared to controls which reached a cut-off point of 25% yellowing at 21 days. A previous study showed that polyethylene bags (HDP) enhance the efficiency of the post-harvest quality of broccoli (29, 35). Toivonen (86) stated that broccoli heads with wrapped packaging contributed to the best retention of firmness and the minimum loss of water independent of storage temperature.

#### Phytochemicals

Tables 6, 7 revealed that chemically treated materials had a significant effect on nutrient retention which kept the phytochemicals intact in broccoli heads during the storage period. Among the treatment combinations, the maximum and appreciable amount of nutrients, viz., protein (3.17%), fat (0.4371%), carbohydrates (5.15%), vitamin C (81.75 mg/100 g), antioxidants (71.75 mg/100 g), and total phenols (39.85 mg/100 g) were retained intact in T_5_ × HDP up to 24 days which were 4.80, 0.091, 1.90, 6.84, 0.97, and 2.88%, respectively lower than the fresh broccoli heads (Table 2). Whereasthe minimum amount of protein (1.87%), fat (0.3671%), carbohydrates (2.46%), vitamin C (53.01 mg/100 g), antioxidants (45.31 mg/100 g), and phenols (20.31 mg/100 g) were retained in T_1_ × con. Broccoli heads under control treatment at 11 days of storage duration, contents of these phytochemicals deteriorated by 44.35, 16.36, 53.6, 40.11, 38.39, and 50.6%, respectively, compared to the fresh ones (Table 2).

**Table 6:** Effects of pre-harvest foliar application of mineral nutrient sources and post-harvest packaging and chemical treatments on nutrient contents broccoli heads under different storage temperatures at the maximum shelf-life stage.

**Treatment combination**	**Protein (%)**		**Fat (%)**		**Carbohydrates (%)**	
	**At room temperature condition (14-22**°**C)**	**In cold storage condition (4**°**C)**	**At room temperature condition (14-22**°**C)**	**In cold storage condition (4**°**C)**	**At room temperature condition (14-22**°**C)**	**In cold storage condition (4**°**C)**
T_1_ × LDP	2.23 ± 0.01bc	2.38 ± 0.02c	0.3737 ± 0.03c	0.3823 ± 0.02c	2.46 ± 0.01cd	2.85 ± 0.01c
T_2_ × LDP	2.54 ± 0.05b	2.79 ± 0.06b	0.4253 ± 0.06ab	0.4301 ± 0.07a	3.59 ± 0.09b	4.05 ± 0.03abc
T_3_ × LDP	3.04 ± 0.03ab	3.27 ± 0.04ab	0.4243 ± 0.04ab	0.4310 ± 0.06a	3.98 ± 0.02abc	4.27 ± 0.09abc
T_4_ × LDP	3.15 ± 0.08ab	3.49 ± 0.09a	0.4060 ± 0.02abc	0.4126 ± 0.01abc	2.92 ± 0.08c	3.25 ± 0.06bc
T_5_ × LDP	2.85 ± 0.04b	3.15 ± 0.05ab	0.4307 ± 0.01a	0.4359 ± 0.04a	4.31 ± 0.03ab	5.09 ± 0.07a
T_1_ × HDP	2.36 ± 0.03bc	2.44 ± 0.03c	0.3753 ± 0.05c	0.3811 ± 0.01c	2.60 ± 0.07c	2.75 ± 0.08c
T_2_ × HDP	2.61 ± 0.07b	2.80 ± 0.08b	0.4279 ± 0.04ab	0.4317 ± 0.2a	3.76 ± 0.4b	4.09 ± 0.04abc
T_3_ × HDP	3.10 ± 0.09ab	3.29 ± 0.02ab	0.4271 ± 0.03ab	0.4341 ± 0.9a	4.08 ± 0.1abc	4.37 ± 0.09abc
T_4_ × HDP	3.22 ± 0.08a	3.51 ± 0.09a	0.4069 ± 0.07abc	0.4141 ± 0.4abc	3.13 ± 0.04bc	3.31 ± 0.05bc
T_5_ × HDP	2.92 ± 0.05ab	3.17 ± 0.06ab	0.4315 ± 0.08a	0.4371 ± 0.9a	4.69 ± 0.02a	5.15 ± 0.07a
T_1_ × ESP	2.19 ± 0.02c	2.27 ± 0.03cd	0.37205 ± 0.02c	0.3749 ± 0.04c	2.87 ± 0.07c	2.72 ± 0.04c
T_2_ × ESP	2.53 ± 0.07b	2.73 ± 0.08b	0.4244 ± 0.06ab	0.4283 ± 0.01ab	3.72 ± 0.09b	4.01 ± 0.06abc
T_3_ × ESP	3.05 ± 0.06ab	3.27 ± 0.07ab	0.4229 ± 0.02a	0.4293 ± 0.9ab	4.02 ± 0.01abc	4.26 ± 0.04abc
T_4_ × ESP	3.17 ± 0.09ab	3.46 ± 0.02a	0.4043 ± 0.07abc	0.4119 ± 0.4abc	3.10 ± 0.07bc	3.22 ± 0.02bc
T_5_ × ESP	3.01 ± 0.07ab	3.02 ± 0.08ab	0.4303 ± 0.09a	0.4359 ± 0.03a	4.47 ± 0.06ab	5.01 ± 0.07ab
T_1_× AA	2.14 ± 0.04c	2.15 ± 0.05cd	0.3717 ± 0.04c	0.3694 ± 0.07d	2.36 ± 0.03d	2.59 ± 0.05c
T_2_ × AA	2.46 ± 0.03bc	2.69 ± 0.04bc	0.4227 ± 0.03ab	0.4226 ± 0.1ab	3.53 ± 0.07b	3.85 ± 0.07b
T_3_ × AA	2.95 ± 0.07ab	3.23 ± 0.08ab	0.4240 ± 0.05ab	0.4264 ± 0.08ab	3.78 ± 0.06b	4.08 ± 0.09abc
T_4_ × AA	3.05 ± 0.06ab	2.88 ± 0.07b	0.4017 ± 0.02abc	0.4089 ± 0.06ab	2.79 ± 0.01c	3.11 ± 0.04bc
T_5_ × AA	2.95 ± 0.02ab	3.01 ± 0.03ab	0.4339 ± 0.07a	0.4346 ± 0.04a	4.17 ± 0.03ab	4.91 ± 0.04a
T_1_ × Con	2.11 ± 0.04c	1.87 ± 0.05d	0.3667 ± 0.09cd	0.3671 ± 0.06d	2.21 ± 0.07d	2.46 ± 0.09d
T_2_ × Con	2.45 ± 0.01bc	2.50 ± 0.02bc	0.4186 ± 0.07abc	0.4145 ± 0.01abc	3.21 ± 0.06bc	3.7 ± 0.04b
T_3_ × Con	2.81 ± 0.08ab	2.96 ± 0.09ab	0.4205 ± 0.01ab	0.4218 ± 0.02ab	3.73 ± 0.01b	3.91 ± 0.02b
T_4_× Con	3.05 ± 0.02ab	2.76 ± 0.03b	0.3977 ± 0.04abc	0.4011 ± 0.04b	2.77 ± 0.03c	2.84 ± 0.07c
T_5_ × Con	2.69 ± 0.04b	2.83 ± 0.05b	0.4271 ± 0.09ab	0.4317 ± 0.09a	4.09 ± 0.08abc	4.51 ± 0.02abc
Level of significance	^**^	^**^	^**^	^**^	^**^	^**^

T_1_ = control, T_2_ = B, T_3_ = Zn, T_4_ = Mo, and T_5_ = B + Zn + Mo, 0.40%, respectively. LDP = low-density polyethylene (35 μm) bag, HDP = High-density polyethylene (15 μm) vacuum pack, ESP = 2% eggshell powder solution, AA = 2% ascorbic acid solution, and Con = control condition. ^**^Significant level at 1%, level of significance, in a column, means having a similar letter(s) are not significantly different.

**Table 7:** Effects of pre-harvest foliar application of mineral nutrient sources and post-harvest packaging and chemical treatments on nutrient contents broccoli heads under different storage temperatures at the maximum shelf-life stage.

**Treatment combination**	**Vitamin C (mg/100 g)**		**Antioxidants (mg/100 g)**		**Phenols (mg/100 g)**	
	**At room temperature condition (14-22**°**C)**	**In cold storage condition (4**°**C)**	**At room temperature condition (14-22**°**C)**	**In cold storage condition (4**°**C)**	**At room temperature condition (14-22**°**C)**	**In cold storage condition (4**°**C)**
T_1_ × LDP	54.10 ± 0.02d	60.04 ± 0.06d	44.05 ± 0.06ef	51.71 ± 0.08e	20.40 ± 0.02f	24.14 ± 0.08de
T_2_ × LDP	71.35 ± 0.04abc	74.02 ± 0.09abc	57.25 ± 0.07c	64.57 ± 0.04b	31.43 ± 0.06b	35.21 ± 0.01ab
T_3_ × LDP	73.35 ± 0.07abc	76.23 ± 0.01ab	59.75 ± 0.01bc	67.32 ± 0.02ab	33.29 ± 0.01abc	37.52 ± 0.4a
T_4_ × LDP	66.13 ± 0.03bc	70.22 ± 0.03bcd	52.36 ± 0.04d	60.76 ± 0.1cd	29.32 ± 0.04c	31.36 ± 0.6cd
T_5_ × LDP	75.72 ± 0.01ab	79.69 ± 0.06a	64.63 ± 0.07ab	70.56 ± 0.03a	35.65 ± 0.09ab	38.69 ± 0.07b
T_1_ × HDP	57.03 ± 0.06cd	62.81 ± 0.01d	47.16 ± 0.2e	54.19 ± 0.02de	23.26 ± 0.04e	25.33 ± 0.01de
T_2_ × HDP	74.43 ± 0.08ab	76.47 ± 0.05ab	59.19 ± 0.8bc	65.82 ± 0.07abc	33.38 ± 0.06ab	36.78 ± 0.07bc
T_3_ × HDP	76.39 ± 0.05a	78.405 ± 0.1ab	61.91 ± 0.6b	68.82 ± 0.03ab	35.41 ± 0.03ab	38.86 ± 0.3b
T_4_ × HDP	69.06 ± 0.09b	73.16 ± 0.8abc	55.87 ± 0.02bc	62.82 ± 0.07c	31.57 ± 0.07b	33.31 ± 0.7c
T_5_ × HDP	77.63 ± 0.03a	81.75 ± 0.2a	66.49 ± 0.07a	71.75 ± 0.01a	37.33 ± 0.06a	39.85 ± 0.04a
T_1_ × ESP	52.04 ± 0.1d	57.28 ± 0.01e	41.98 ± 0.03d	49.94 ± 0.09ef	19.20 ± 0.04fg	23.61 ± 0.07de
T_2_ × ESP	69.45 ± 0.5b	70.97 ± 0.07bcd	56.17 ± 0.08cd	62.35 ± 0.01c	29.90 ± 0.02c	34.7 ± 0.02bc
T_3_ × ESP	72.10 ± 0.06abc	74.44 ± 0.03ab	58.24 ± 0.06c	65.21 ± 0.08abc	31.96 ± 0.07b	36.62 ± 0.03bc
T_4_ × ESP	64.55 ± 0.08bc	67.51 ± 0.2cd	51.18 ± 0.03d	59.615 ± 0.05cd	27.79 ± 0.06cd	30.33 ± 0.05cd
T_5_ × ESP	73.15 ± 0.04ab	77.81 ± 0.3ab	62.21 ± 0.04ab	67.331 ± 0.07ab	33.62 ± 0.01abc	36.29 ± 0.01b
T_1_× AA	49.91 ± 0.07de	55.10 ± 0.08c	40.75 ± 0.03d	47.21 ± 0.02f	16.29 ± 0.03g	21.09 ± 0.08e
T_2_ × AA	67.38 ± 0.01ab	67.29 ± 0.1cd	55.06 ± 0.07cd	60.29 ± 0.05cd	27.22 ± 0.04cd	32.86 ± 0.04c
T_3_ × AA	70.19 ± 0.06a	71.41 ± 0.04c	57.07 ± 0.01c	63.26 ± 0.09bc	29.16 ± 0.07c	34.43 ± 0.06bc
T_4_ × AA	61.98 ± 0.09c	63.36 ± 0.09abc	49.05 ± 0.06e	56.19 ± 0.03a-d	25.25 ± 0.01d	29.27 ± 0.07d
T_5_ × AA	73.66 ± 0.02ab	73.75 ± 0.07abc	65.43 ± 0.04a	66.59 ± 0.4ab	33.96 ± 0.03ab	34.77 ± 0.03bc
T_1_ × Con	46.83 ± 0.04f	53.01 ± 0.02f	37.26 ± 0.06g	45.31 ± 0.7fg	15.41 ± 0.04gh	20.31 ± 0.06e
T_2_ × Con	65.43 ± 0.06bc	65.40 ± 0.08ab	53.17 ± 0.02d	57.96 ± 0.09d	25.32 ± 0.09d	31.81 ± 0.08c
T_3_ × Con	68.21 ± 0.02b	70.25 ± 0.3a	55.09 ± 0.07cd	61.09 ± 0.04cd	27.41 ± 0.04cd	33.42 ± 0.04c
T_4_× Con	58.33 ± 0.07cd	61.11 ± 0.04d	47.16 ± 0.03e	54.21 ± 0.03de	22.75 ± 0.01ef	28.01 ± 0.06d
T_5_ × Con	68.33 ± 0.03b	70.36 ± 0.07a	60.03 ± 0.07b	61.75 ± 0.04b	28.45 ± 0.04c	33.85 ± 0.01bc
Level of significance	^**^	^**^	^**^	^**^	^**^	^**^

T_1_ = control, T_2_ = B, T_3_ = Zn, T_4_ = Mo, and T_5_ = B + Zn + Mo, 0.40%, respectively. LDP = Low-density polyethylene (35 μm) bag, HDP = High-density polyethylene (15 μm) vacuum pack, ESP = 2% eggshell powder solution, AA = 2% ascorbic acid solution, and Con = control condition. ^**^Significant level at 1%, level of significance, In a column, means having a similar letter(s) are not significantly different.

Similarly, when broccoli heads were stored at room temperature (14–22°C with RH 60–65%), the various nutrients, viz., protein (2.92%), fats (0.4315%), carbohydrates (4.69%), vitamin C (77.63 mg/100 g), antioxidants (66.49 mg/100 g), and total phenols (37.33 mg/100 g) retained intact up to 7 days in T_5_ × HDP which were 12.31%, 1.37%, 10.67%, 11.53%, 8.23%, and 9.02%, respectively lower than compared to the fresh broccoli heads (Tables 2, 6, 7). Hasan et al. (87) reported a decline in ascorbic acid of fresh pointed gourd under cold storage up to shelf-life. Whereas, the minimum amount of nutrients, viz., protein (2.11%), fats (0.3667%), carbohydrates (2.21%), vitamin C (46.83 mg/100 g), antioxidants (37.26 mg/100 g), and total phenols (15.41 mg/100 g) were retained intact in T_1_ × con. at room temperature for up to 2 days, which were 37.2%, 16.45%, 8.38%, 47.09%, 49.33%, and 62.52%, respectively lower than fresh broccoli heads (Tables 2, 6, 7). It was evident from the results in Tables 6, 7 that the HDP vacuum pack (15 μm) was meaningfully effective for maintaining the broccoli heads' quality by keeping its nutrients intact at the highest shelf-life both at cold storage and room temperature conditions. Commination of pre-harvest nutrients sprays such as Mo, B, Zn, and Mo + B + Zn and post-harvest treatments, such as eggshells (2%) and ascorbic acids (2%) demonstrated medium-to-low interaction effects for protein, fat, carbohydrates, vitamin C, antioxidants, and total phenols. HDP is a viable post-harvest packaging technology to extend the shelf-life of broccoli which slows down the respiration rate and ethylene production, intact color, texture, flavor, and nutritive values. These findings are consistent with the findings of Chingtham and Banik (29). According to Shirley (88), frozen broccoli losses ~50–55% of its vitamin C, while storing broccoli at a minimum of 4°C helps to retain vitamin C.

### Economic performance

The economic performance of broccoli varied due to different treatment combinations as influenced by the foliar application of different mineral nutrients. The results revealed that the maximum production cost of BDT 114648 ha^−1^ was recorded in B + Zn + Mo followed by Mo (BDT 111734 ha^−1^) (Table 8). The lowest production cost (BDT 107620 ha^−1^) was noted in control. The lowest gross return (BDT 420300 ha^−1^) and net return (BDT 305652 ha^−1^) were documented in B + Zn + Mo, followed by B with a gross return of BDT 398250 ha^−1^, the net return of BDT 288985 ha^−1^. Whereas the lowest gross return (BDT 339600 ha^−1^) and net return (BDT 231980 ha^−1^) were noted in control. The lowest benefit–cost ratio (BCR) of 3.67 was documented in B + Zn + Mo followed by B (BCR 3.64) and Zn (BCR 3.57). The lowest BCR (3.16) was found in the control. These findings conform with a few previous studies (28, 58, 59, 89).

**Table 8:** Economic performance of broccoli production by pre-harvest foliar application effects of mineral nutrients.

**Treatment**	**Marketable head yield (t ha^−1^)**	**Cost of production (BDT ha^−1^)**	**Gross returns (BDT ha^−1^)**	**Net returns (BDT ha^−1^)**	**Benefit-cost ratio (BCR)**
T_1_	22.64	109760	339600	231980	3.16
T_2_	26.55	111734	398250	288985	3.64
T_3_	26.1		391500	281740	3.57
T_4_	25.39		380850	269116	3.41
T_5_	28.02	114648	420300	305652	3.67

Sale rate of broccoli (BDT15/kg). T_1_ = control, T_2_ = B, T_3_ = Zn, T_4_ = Mo, and T_5_ = B + Zn + Mo, 0.40%, respectively.

## Conclusion

The present study confirmed the maximum marketable head yield of 28.02 t ha^−1^, a gross return of BDT 420300 ha^−1^, a net return of BDT 305652 ha^−1^, and a maximum benefit–cost ratio (BCR) of 3.67. Combined nutrient spray during the growing of broccoli efficiently improves physicochemical attributes, viz., compactness, color, texture, carbohydrates, fats, energy, antioxidants, vitamin C, and total phenol content in broccoli heads. Furthermore, combined nutrient spray and HDP vacuum pack (15 μm) during post-harvest also established a shelf-life of 24.55 days at cold storage (RH 90–95% and 4°C) and 7.05 days at room temperature (RH 60–65% and 14–22°C) conditions. HDP vacuum pack (15 μm) was suggestively effective in maintaining the quality of broccoli heads and keeping nutrients at the highest shelf-life stage both at cold storage and room temperature conditions. Therefore, we recommend the application of combined nutrient elements B + Zn + Mo and HDP (15 μm) vacuum packaging for the end users to obtain optimum marketable head yield for anticipating physicochemical attributes and maximum shelf-life of broccoli. Though this study was accomplished in an arbitrarily selected location, we recommend multilocation trails (MLTs) in the agro-ecological zones (AEZs) to explore the genotype and environmental interaction for further confirmation of the findings.

## Data availability statement

The original contributions presented in the study are included in the article/Supplementary material, further inquiries can be directed to the corresponding authors.

## Author contributions

MB and ST: conceptualization. ST and US: methodology, original draft preparation, and software. ST, US, SE, ZO, RM, and KG: formal analysis. ST: data curation and investigation. US, SE, ZO, RM, and KG: writing of review and editing. US: visualization. MB: supervision. US, SE, RM, and KG: validation. All authors contributed to the article and approved the submitted version.

## References

[1] Chakrabarty T Sarker U Hasan M Rahman MM. Variability in mineral compositions, yield and yield contributing traits of stem amaranth (*Amaranthus lividus*). Genetika. (2018) 50:995–1010. 10.2298/GENSR1803995C

[2] Sarker U Azam MG Talukder MZA. Genetic variation in mineral profiles, yield contributing agronomic traits, and foliage yield of stem amaranth. Genetika. (2022) 54:91–108. 10.2298/GENSR2201091S

[3] Dola DB Mannanm MA Sarker U Mamun MAA Islam T Ercisli S. Nano-iron oxide accelerates growth, yield, and quality of Glycine max seed in water deficits. Front Plant Sci. (2022) 13:992535. 10.3389/fpls.2022.992535 36160973 PMC9500458

[4] Hossain MN Sarker U Raihan MS Al-Huqail AA Siddiqui MH Oba S. Influence of salinity stress on color parameters, leaf pigmentation, polyphenol and flavonoid contents, and antioxidant activity of *Amaranthus lividus* leafy vegetables. Molecules. (2022) 27:1821. 10.3390/molecules27061821 35335185 PMC8955103

[5] Hassan J Rajib MMR Sarker U. Optimizing textile dyeing wastewater for tomato irrigation through physiochemical, plant nutrient uses and pollution load index of irrigated soil. Sci Rep. (2022) 12:10088. 10.1038/s41598-022-11558-1 35710771 PMC9203507

[6] Sarker U Rabbani MG Oba S Eldehna WM Al-Rashood ST Mostafa NM. Phytonutrients, colorant pigments, phytochemicals, and antioxidant potential of orphan leafy Amaranthus species. Molecules. (2022) 27:2899. 10.3390/molecules27092899 35566250 PMC9101061

[7] Sarker U Oba S Alsanie WF Gaber A. Characterization of phytochemicals, nutrients, and antiradical potential in slim amaranth. Antioxidants. (2022) 11:1089. 10.3390/antiox11061089 35739986 PMC9219808

[8] Sarker U Oba S. Antioxidant constituents of three selected red and green color Amaranthus leafy vegetable. Sci Rep. (2019) 9:18233. 10.1038/s41598-019-52033-8 31796754 PMC6890792

[9] Sarker U Oba S. Leaf pigmentation, its profiles and radical scavenging activity in selected Amaranthus tricolor leafy vegetables. Sci Rep. (2020) 10:18617. 10.1038/s41598-020-66376-0 33122663 PMC7596228

[10] Sarker U Oba S. Color Attributes, betacyanin, and carotenoid profiles, bioactive components, and radical quenching capacity in selected *Amaranthus gangeticus* leafy vegetables. Sci Rep. (2021) 11:11559. 10.1038/s41598-021-91157-8 34079029 PMC8172918

[11] Sarker U Oba S. Protein, dietary fiber, minerals, antioxidant pigments and phytochemicals, and antioxidant activity in selected red morph Amaranthus leafy vegetable. PLoS ONE. (2019) 14:222517. 10.1371/journal.pone.0222517 31830064 PMC6907799

[12] Sarker U Oba S. Nutraceuticals, phytochemicals, and radical quenching ability of selected drought-tolerant advance lines of vegetable amaranth. BMC Plant Biol. (2020) 20:564. 10.1186/s12870-020-02780-y 33317465 PMC7737358

[13] Sarker U Islam MT Rabbani MG Oba S. Variability, heritability and genetic association in vegetable amaranth (A. tricolor). Span J Agric Res. (2015) 13:1–8. 10.5424/sjar/2015132-684316736385

[14] Sarker U Lin YP Oba S Yoshioka Y Ken H. Prospects and potentials of underutilized leafy Amaranths as vegetable use for health promotion. Plant Physiol Biochem. (2022) 182:104–23. 10.1016/j.plaphy.2022.04.011 35487123

[15] Hansen M Møller PR Sørensen H Trejo MC. Glucosinolates in broccoli stored under controlled atmosphere. J Am Soc Horticult Sci. (1995) 120:1069–74. 10.21273/JASHS.120.6.1069

[16] Farnham MW Stephenson KK Fahey JW. Glucoraphanin level in broccoli seed is largely determined by genotype. HortScience HortSci. (2005) 40:50–3. 10.21273/HORTSCI.40.1.5015113170

[17] Martins T Oliveira PA Pires MJ Neuparth MJ Lanzarin G Félix L. Effect of a sub-chronic oral exposure of broccoli (*Brassica oleracea L.* Var Italica) by-products flour on the physiological parameters of fvb/n mice: a pilot. Study Foods. (2022) 11:120. 10.3390/foods11010120 35010245 PMC8750293

[18] Cartea ME Francisco M Soengas P Velasco P. Phenolic compounds in brassica vegetables. Molecules. (2011) 16:251–80. 10.3390/molecules16010251 21193847 PMC6259264

[19] Faller ALK Fialho E. The antioxidant capacity and polyphenol content of organic and conventional retail vegetables after domestic cooking. Food Res Int. (2009) 42:210–5. 10.1016/j.foodres.2008.10.009

[20] Nagraj GS Anita C Swarna J Amit KJ. Nutritional Composition and Antioxidant Properties of Fruits and Vegetables. Dublin: Academic Press (2020), 5–17.

[21] FAOSTAT. Food and Agriculture Organization of the United Nations, Statistics Division, Corporate Statistical Database (FAOSTAT). (2022), Rome.

[22] Ahirwar CS Nath R. Organic broccoli farming: a step towards doubling farmer‘s income. Res Today. (2020) 2:47–50.

[23] Mal D Chatterjee R Nimbalkar KH. Effect of vermicompost and inorganic fertilizers on growth, yield and quality of sprouting broccoli (*Brassica oleracea* L. var Italica Plenck). Intl J Bio-Res Stress Manage. (2015) 5:507–12. 10.5958/0976-4038.2014.00606.X

[24] Moniruzzaman M Rahman SM Rahman MA. Effect of boron on yield and hollow stem of cauliflower and broccoli. J Soil Sci. (2007) 1:24–9.

[25] Patel A Maji S Meena KR Malviya NK. Use of boron and molybdenum to improve broccoli production. J Crop Weed. (2017) 13:20–4.20659758

[26] Ain Q Ayub G Ilyas M Ahmad M Begum F Man L. Response of broccoli to foliar application of zinc and boron concentrations. Pure Appl Biol. (2016) 5:841–6. 10.19045/bspab.2016.5010533139772

[27] Bakhtiar M Farooq M Ahmed S Ilyas N Khan I Saboor A. Influence of sulfur and boron on the growth and yield of broccoli. Intl J Environ Agric Res. (2018) 4:9–16.

[28] Singh V Singh AK Singh S Kumar A Mohrana DP. Impact of foliar spray of micronutrients on growth, yield and quality of broccoli (*Brassica oleracea* var. Italica) cv Pusa KTS-1. Pharma Innova J. (2018) 7:99–101.

[29] Chingtham C Banik A. Studies on effectiveness of packaging on storability of broccoli cv. Aishwarya Intl J Chem Stud. (2019) 7:5112–8.

[30] Raseetha S Nadirah S. Effect of different packaging materials on quality of fresh-cut broccoli and cauliflower at chilled temperature. Intl Food Res J. (2018) 25:1559–65.

[31] Rao DVS Shivashankara KS. Individual shrink wrapping extends the storage life and maintains the antioxidants of mangoes (cvs ‘Alphonso' and ‘Baganapalli') stored at 8°C. J Food Sci Technol. (2015) 52:4351–9. 10.1007/s13197-014-1468-6 26139900 PMC4486553

[32] Jadhav PB Gurav NP More DB. Extending the shelf- life of broccoli cv. ‘Green Majic' using a cold room (Eco-frost). Intl J Agric Sci. (2018) 10:7087–91.

[33] Sharma P. Effect of foliar spray of micronutrients on growth, yield and quality of broccoli (Brassica oleracea var. Italica) cv. Pusa KTS-1. (Doctoral dissertation, Institution of Agricultural Sciences, Banaras Hindu University), India (2012).

[34] Chowdhury R Sikder S. Study the manifestation of growth and yield attributes of Broccoli through application of boron, molybdenum, zinc and their combination treatments in Terai agro-ecological region of West Bengal. Curr Agric Res. (2017) 5:366–70. 10.12944/CARJ.5.3.16

[35] Beer D Crouch T. Packaging in the maintenance of postharvest keeping quality of 'Parthenon' broccoli during long term storage and subsequent shelf-life. Acta Hortic. (2013) 1007:65–72. 10.17660/ActaHortic.2013.1007.4

[36] Thakur RJ Shaikh H Gat Y Waghmare RB. Effect of calcium chloride extracted from eggshell in maintaining quality of selected fresh-cut fruits. Int J Recycl Org Waste Agric. (2019) 8:27–36. 10.1007/s40093-019-0260-z

[37] Singh J Mirza A. Influence of ascorbic acid application on quality and storage life of fruits. Int J Curr Microbiol App Sci. (2018) 7:4319–28. 10.20546/ijcmas.2018.707.503

[38] Bangladesh Agricultural Research Council (BARC). (2018). FRG-2018 (Fertilizer Recommendation Guide-2018). Dhaka: Bangladesh Agricultural Research Council (BARC). p. 220–5.

[39] Ranganna S. Handbook of Analysis and Quality Control for Fruits and Vegetables Products. New Delhi: Tata Mc Graw Hill Publication Co. Ltd. (1986), 497–529.

[40] Sarker U Hossain MN Iqbal MA Oba S. Bioactive components and radical scavenging activity in selected advance lines of salt-tolerant vegetable amaranth. Front Nutr. (2020) 7:587257. 10.3389/fnut.2020.587257 33330589 PMC7734134

[41] Sarker U Oba S Daramy MA. Nutrients, minerals, antioxidant pigments and phytochemicals, and antioxidant capacity of the leaves of stem amaranth. Sci Rep. (2020) 10:3892. 10.1038/s41598-020-60252-7 32127553 PMC7054523

[42] Sarker U Oba S. Nutrients, minerals, pigments, phytochemical, and radical scavenging activity in *Amaranthus blitum* leafy vegetable. Sci Rep. (2020) 10:3868. 10.1038/s41598-020-59848-w 32123184 PMC7051949

[43] Sarker U Oba S. Nutritional and bioactive constituents and scavenging capacity of radicals in *Amaranthus hypochondriacus*. Sci Rep. (2020) 10:19962. 10.1038/s41598-020-71714-3 33203902 PMC7673121

[44] Sarker U Oba S. Response of nutrients, minerals, antioxidant leaf pigments, vitamins, polyphenol, flavonoid and antioxidant activity in selected vegetable amaranth under four soil water content. Food Chem. (2018) 252:72–83. 10.1016/j.foodchem.2018.01.097 29478565

[45] Sarker U Oba S Ercisli S Assouguem A Alotaibi A Ullah R. Bioactive phytochemicals and quenching activity of radicals in selected drought-resistant Amaranthus tricolor vegetable amaranth. Antioxidants. (2022) 11:578. 10.3390/antiox11030578 35326227 PMC8944989

[46] Sarker U Iqbal MA Hossain MN Oba S Ercisli S Muresan CC. Colorant pigments, nutrients, bioactive components, and antiradical potential of danta leaves (*Amaranthus lividus*). Antioxidants. (2022) 11:1206. 10.3390/antiox11061206 35740102 PMC9219785

[47] Sarker U Oba S. Drought stress enhances nutritional and bioactive compounds, phenolic acids and antioxidant capacity of Amaranthus leafy vegetable. BMC Plant Biol. (2018) 18:258. 10.1186/s12870-018-1484-1 30367616 PMC6203965

[48] Sarker U Oba S. Nutraceuticals, antioxidant pigments, and phytochemicals in the leaves of *Amaranthus spinosus* and *Amaranthus viridis* weedy species. Sci Rep9. (2019) 20413. 10.1038/s41598-019-50977-5 31892700 PMC6938518

[49] Sarker U Oba S. Salinity stress enhances color parameters, bioactive leaf pigments, vitamins, polyphenols, flavonoids and antioxidant activity in selected Amaranthus leafy vegetables. J Sci Food Agric. (2019) 99:2275–84. 10.1002/jsfa.9423 30324618

[50] Sarker U Hossain MM Oba S. Nutritional and antioxidant components and antioxidant capacity in green morph Amaranthus leafy vegetable. Sci Rep10. (2020) 1336. 10.1038/s41598-020-57687-3 31992722 PMC6987210

[51] Sarker U Islam MT Oba S. Salinity stress accelerates nutrients, dietary fiber, minerals, phytochemicals and antioxidant activity in Amaranthus tricolor leaves. PLoS ONE. (2018) 13:0206388. 10.1371/journal.pone.0206388 30383779 PMC6211690

[52] Sarker U Ercisli S. Salt Eustress Induction in Red Amaranth (*Amaranthus gangeticus*) augments nutritional, phenolic acids and antiradical potential of leaves. Antioxidants. (2022) 11:2434. 10.3390/antiox11122434 36552642 PMC9774578

[53] Sarker U Hossain MN Oba S Ercisli S Marc RA Golokhvast KS. Salinity stress ameliorates pigments, minerals, polyphenolic profiles, and antiradical capacity in Lalshak. Antioxidants. (2023) 12:173. 10.3390/antiox12010173 36671036 PMC9855230

[54] Sarker U Oba S. The response of salinity stress-induced a tricolor to growth, anatomy, physiology, non-enzymatic and enzymatic antioxidants. Front Plant Sci. (2020) 11:559876. 10.3389/fpls.2020.559876 33178233 PMC7596248

[55] AOAC. Official Methods AOAC of Analysis of Association of Official Analytical Chemists, 15th Edn. Arlington, VA: AOAC (1990), 1–50.

[56] Singh GS Sarvanan KSR Jalam SR Bhanwar L. Effect of different micronutrients on plant growth, yield and flower bud quality of broccoli (*Brassica oleracea* var. Italica) cv. Green Bud Intl J Adv Res. (2016) 4:2018–43. 10.21474/IJAR01/1686

[57] Bairwa PL Dixit A Sahu MK. Effect of different micronutrients on growth and yield of cauliflower (*Brassica oleracea* var. Botrytis L) cv Pusa Sharad. Int J Chem Stud. (2021) 9:3084–9. 10.22271/chemi.2021.v9.i1ak.11627

[58] Xaxa S Praveen C Radhelal D Preeti T Mithlesh G Sunny AT. Effect of different micronutrients on plant growth and yield of broccoli (Brassica oleracea var. Italica). Intl J Chem Stud. (2018) 6:979–82.25623939

[59] Hatwar GP Gondane SM Urkude SM Gahukar OV. Effect of micronutrients on growth and yield of chilli. J Soils Crops. (2003) 13:123–5.29605647

[60] Ahmed ME Elzaawely AA El-Sawy MB. Effect of the foliar spraying with molybdenum and magnesium on vegetative growth and head yields in Cauliflower (Brassica oleracea var. Botrytis). World J Agril Sci. (2011) 7:149–56.

[61] Dhakal D Shah SC Gautam DM Yadav RM. Response of cauliflower (Brassica oleracea var. Botrytis) to the application of boron and phosphorus in the soils of Rupandehi District Nepal. Agric Res J. (2009) 9:56–66. 10.3126/narj.v9i0.11642

[62] Mohamed ME Abdel W Mahmoud M Mohamed BIE Aditya P. Pre-harvest foliar application of mineral nutrients to retard chlorophyll degradation and preserve bio-active compounds in broccoli. Agronomy. (2019) 9:711. 10.3390/agronomy9110711

[63] Alloway BJ. Micronutrients and Crop Production: An Introduction in Micronutrients Deficiencies in Global Crop Production. Heidelberg: Springer (2008), 353.

[64] Broadley M Brown PIC Rengel Z Zhao F. Function of Nutrients: Micronutrients. In:P Marschner, editor Marschner's Mineral Nutrition of Higher Plants, Amsterdam: Elsevier (2012), 191–248.

[65] Liu H Gan W Rengel Z Zhao P. Effects of zinc fertilizer rate and application method on photosynthetic characteristics and grain yield of summer maize. J Soil Sci Plant Nutr. (2016) 16:550–62. 10.4067/S0718-9516201600500004527315006

[66] Aghdam MS Hassanpouraghdam MB Paliyath G Farmani B. The language of calcium in postharvest life of fruits, vegetables and flowers. Sci Hortic. (2012) 144:102–15. 10.1016/j.scienta.2012.07.007

[67] Duffy B. Zinc and plant disease. In:Datnoff WH Elmer DM Huber, editors *Mineral Nutrition and Plant Disease*. São Paulo, MN: The American Phyto-pathological Society (2007), 155–75.

[68] Burnell JN. The Biochemistry of Manganese in Plants. In Manganese in Soils and Plants, Proceedings of the International Symposium on ‘Manganese in Soils and Plants' held at the Waite Agricultural Research Institute, The University of Adelaide, Glen Osmond, South Australia. Dordrecht: Springer (1988), 125–137.

[69] Weisany W Raei Y Allahverdipoo KH. Role of some of mineral nutrients in biological nitrogen fixation. Bull Env Pharmacol Life Sci. (2013) 2:77–84.

[70] Ali N Hadi F. CBF/DREB transcription factor genes play role in cadmium tolerance and phytoaccumulation in *Ricinus communis* under molybdenum treatments. Chemosphere. (2018) 208:425–32. 10.1016/j.chemosphere.2018.05.165 29885509

[71] Shen ZG Liang YC Shen K. Effect of boron on the nitrate reductase activity in oilseed rape plants. J Plant Nutr. (1993) 16:1229–39. 10.1080/0190416930936460811757364

[72] Gilani SAQ Basit A Sajid M. Gibberellic acid and boron enhance antioxidant activity, phenolic content, and yield quality in *Pyrus Communis* L. Gesunde Pflanzen. (2021) 73:395–406. 10.1007/s10343-021-00555-5

[73] Sarker U Islam MT Rabbani MG Oba S. Antioxidant leaf pigments and variability in vegetable amaranth. Genetika. (2018) 50:209–20. 10.2298/GENSR1801209S

[74] Sarker U Islam MT Rabbani MG Oba S. Variability in total antioxidant capacity, antioxidant leaf pigments and foliage yield of vegetable amaranth. J Integra Agric. (2018) 17:1145–53. 10.1016/S2095-3119(17)61778-7

[75] Rice-Evans C Miller N Paganga G. Antioxidant properties of phenolic compounds. Trends Plant Sci. (1997) 2:152–9. 10.1016/S1360-1385(97)01018-2

[76] Sarker U Oba S. Phenolic profiles and antioxidant activities in selected drought-tolerant leafy vegetable amaranth. Sci Rep. (2020) 10:18287. 10.1038/s41598-020-71727-y 33106544 PMC7589480

[77] Sarker U Oba S. Polyphenol and flavonoid profiles and radical scavenging activity in selected leafy vegetable *Amaranthus gangeticus*. BMC Plant Biol. (2020) 20:499. 10.1186/s12870-020-02700-0 33138787 PMC7607633

[78] Sarker U Oba S. Augmentation of leaf color parameters, pigments, vitamins, phenolic acids, flavonoids and antioxidant activity in selected A tricolor under salinity stress. Sci Rep. (2018) 8:12349. 10.1038/s41598-018-30897-6 30120319 PMC6098045

[79] Sarker U Oba S. Catalase, superoxide dismutase and ascorbate-glutathione cycle enzymes confer drought tolerance of *A*. tricolor Sci Rep. (2018) 8:16496. 10.1038/s41598-018-34944-0 30405159 PMC6220278

[80] Sarker U Oba S. Drought stress effects on growth, ROS markers, compatible solutes, phenolics, flavonoids and antioxidant activity in *A. tricolor*. Appl Biochem Biotechnol. (2018) 186:999–1016. 10.1007/s12010-018-2784-5 29804177

[81] Šlosár M Mezeyová I Hegedüsová A Andrejiová A Kováčik P Lošák T. Effect of zinc fertilisation on yield and selected qualitative parameters of broccoli. Plant Soil Environ. (2017) 63:282–7. 10.17221/220/2017-PSE

[82] Gugała M Sikorska A. The effect of fertilization with sulphur, boron, and amino acids on the content of glucosinolate in winter rape seeds. Agronomy. (2020) 10:519. 10.3390/agronomy10040519

[83] Ferdousi J Zakaria M Hossain MI Saha M. Shelf-life and economic analysis of broccoli (*Brassica oleracea* var. Italica L) as influenced by nitrogen, phosphorus, potassium and molybdenum. J Sylhet Agril Univ. (2014) 1:29–33.

[84] Roni MS Zakaria MM Rasul GHM Nurealam M Siddiqui M. Effect of temperature on shelf-life and ascorbic acid content of broccoli produced with different combinations of nitrogen level and spacing. Int J Biosci. (2014) 5:81–6. 10.12692/ijb/5.6.81-86

[85] Eason JR Ryan D Page B Watson L Coupe SA. Harvested broccoli Brassica oleracea responds to high carbon dioxide and low oxygen atmosphere by inducing stress-response genes. Postharvest Biol. Technol. (2006) 43:358–65. 10.1016/j.postharvbio.2006.10.001

[86] Toivonen PMA. The effects of storage temperature, storage duration, hydro-cooling and micro-perforated wrap on shelf life of broccoli Brassica oleracea L. Postharvest Biol Technol. (1997) 10:59–65. 10.1016/S0925-5214(97)87275-4

[87] Hassan J Jahan F Rajib MMR Sarker U Miyajima I Ozaki Y. Color and physiochemical attributes of pointed gourd (*Trichosanthes dioica* Roxb.) influenced by modified atmosphere packaging and postharvest treatment during storage. Fron. Plant Sci. (2022) 13:1016324. 10.3389/fpls.2022.1016324 36275589 PMC9583917

[88] Shirley J,. Cold Storage of Broccoli Helps Maintain Its Shelf Life Quality. (2021). Available online at: https://www.medindia.net/news/healthinfocus/cold-storage-of-broccoli-helps-maintain-its-shelf-life-and-quality-148982-1.htm (accessed May 5, 2022).

[89] Prodhan MM Sarker U Hoque MA Biswas MS Ercisli S Assouguem A. A foliar application of GA_3_ stimulates seed production in cauliflower. Agronomy. (2022) 12:1394. 10.3390/agronomy12061394

