# Supplementary material for: Influence of foliar spray and post-harvest treatments on head yield, shelf-life, and physicochemical qualities of broccoli
Source: Front Nutr. 2023 Apr 17;10:1057084. doi: 10.3389/fnut.2023.1057084 (PMC10149915; doi:10.3389/fnut.2023.1057084)
Supplement: Supplementary file 1 [file Data_Sheet_1.docx]

**Supplementary Tables**

**Table S1.** Physical and Chemical properties of the experimental plots soil (0-15 cm depth)

| SL No | Properties | Values obtained | Methods of determination |
| --- | --- | --- | --- |
| 1. Physical properties | | | |
| 1 | Soil texture |  |  |
| 2 | Textural class: loamy soil |  |  |
| 3 | Sand (%) | 17.23 | By International Pippetic method |
| 4 | Sit (%) | 56.72 |  |
| 5 | Clay (%) | 26.05 |  |
| B. Chemical properties | | | |
| 1 | Soil P^H^ | 7.25 | pH meter, based on 1: 2.5 |
| 2 | Salinity | 1.71 dS/m |  |
| 3 | Organic matter | 1.27 |  |
| 4 | Total nitrogen | 0.01% | Micro-kjeldahl method |
| 5 | Available phosphorus | 11.30 µg/g soil | Olsen’s method |
| 6 | Available potassium | 0.03 mili equivalent/100 g soil | Flame photometric method |
| 7 | Sulphur | 1.10 µg/g soil | Spectrophotometric method |
| 8 | Zinc | 0.25 µg/g soil | Atomic Absorption Spectrophotometric method |
| 9 | Boron | 0.20 µg/g soil |  |

**Table S2.** Physical and chemical properties of farm yard manure (FYM)

| Properties | FYM | Methods followed |
| --- | --- | --- |
| 1. Color | Gray to dark | Sensory method |
| 2. Odor | Odorless |  |
| 3. Physical condition | Non-granular | Eye estimation |
| 1. P^H^ | 8.5 | P^H^ Meter, Based on 1:2.5 ratio |
| 2. Moisture (%) | 16.0% | Vacuum Desiccators Method |
| 3. Organic Carbon (%) | 6.35% | Wet Oxidation Method |
| 4. C: N | 8.7: 1 |  |
| 5. Total Nitrogen (N), Weight basis | 0.55% | Micro-Kjeldahl Method |
| 6. Total Phosphorus (P), Weight basis | 0.29% | Spectrophotometric Method |
| 7.Total Potassium (K), Weight basis | 0.54% | Flame photometric Method |
| 8. Total Sulfur (S), weight basis | 0.10% | Spectrophotometric Method |
| 9. Total Zinc (Zn), weight basis | 0.003% | Atomic Absorption Spectrophotometric Method |
| 10. Total Cupper (Cu) weight basis | 0.00% |  |
| 11. Total Pb Weight basis | < 0.01 ppm |  |
| 12. Total Cd Weight basis | < 0.01 ppm |  |
