# Supplementary material for: Influence of foliar spray and post-harvest treatments on head yield, shelf-life, and physicochemical qualities of broccoli
Source: Front Nutr. 2023 Apr 17;10:1057084. doi: 10.3389/fnut.2023.1057084 (PMC10149915; doi:10.3389/fnut.2023.1057084)
Supplement: Supplementary file 2 [file Image_1.pdf]

Supplementary Figure

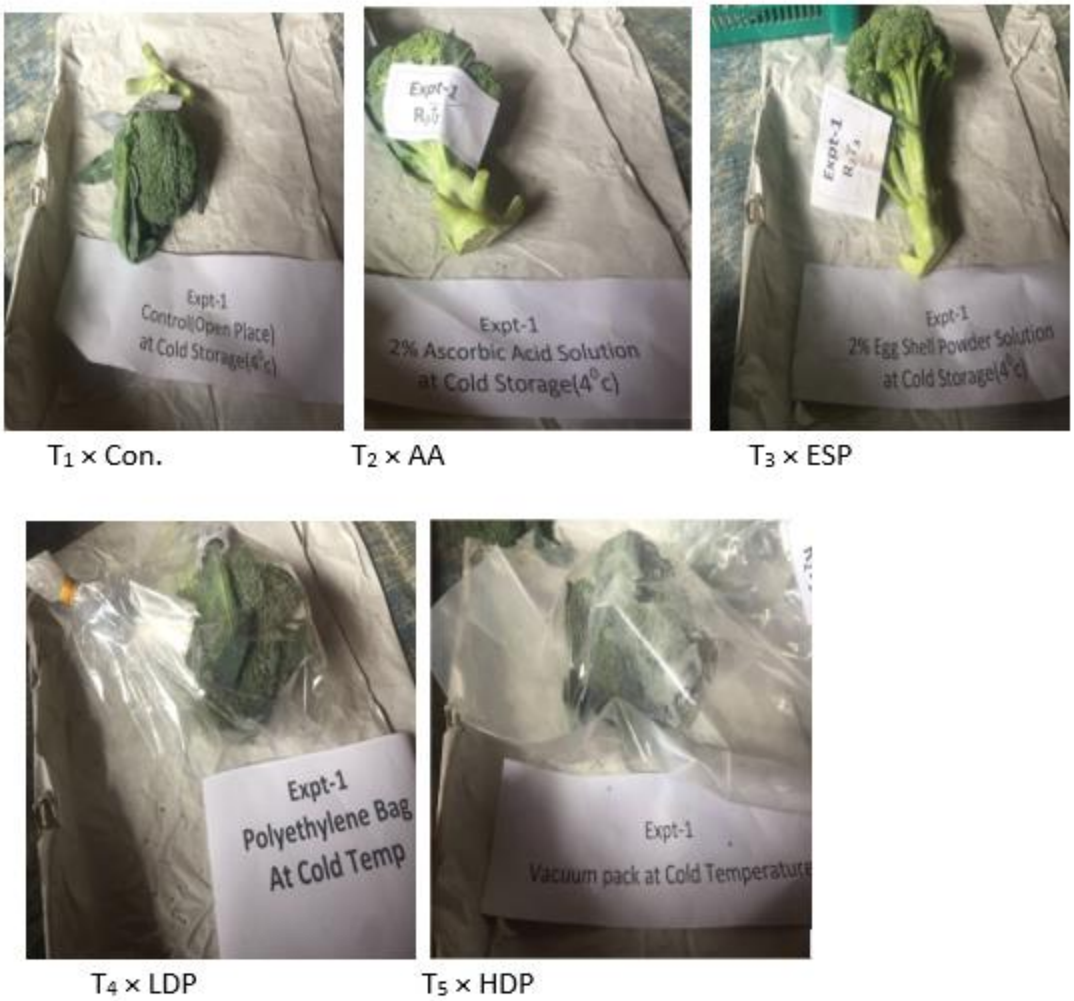

Figure S1. Broccoli heads showing five treatments combinations at cold storage. Con, Control; AA, ascorbic acid; ESP, Eggshell powder; LDP, low-density polyethylene; HDP, High-density polyethylene. T_1 = Control, T_2 = B, T_3 = Zn, T_4 = Mo, and T_5 = B + Zn + Mo, 0.40%, respectively.
